# Temporal Analysis of the Honey Bee Microbiome Reveals Four Novel Viruses and Seasonal Prevalence of Known Viruses, *Nosema*, and *Crithidia*

**DOI:** 10.1371/journal.pone.0020656

**Published:** 2011-06-07

**Authors:** Charles Runckel, Michelle L. Flenniken, Juan C. Engel, J. Graham Ruby, Donald Ganem, Raul Andino, Joseph L. DeRisi

**Affiliations:** 1 Howard Hughes Medical Institute, Bethesda, Maryland, United State of America; 2 Departments of Medicine, Biochemistry and Biophysics, and Microbiology, University of California San Francisco, San Francisco, California, United States of America; 3 Department of Microbiology and Immunology, University of California San Francisco, San Francisco, California, United States of America; 4 Sandler Center for Drug Discovery and Department of Pathology, University of California San Francisco, San Francisco, California, United States of America; Martin-Luther-Universität Halle, Germany

## Abstract

Honey bees (*Apis mellifera*) play a critical role in global food production as pollinators of numerous crops. Recently, honey bee populations in the United States, Canada, and Europe have suffered an unexplained increase in annual losses due to a phenomenon known as Colony Collapse Disorder (CCD). Epidemiological analysis of CCD is confounded by a relative dearth of bee pathogen field studies. To identify what constitutes an abnormal pathophysiological condition in a honey bee colony, it is critical to have characterized the spectrum of exogenous infectious agents in healthy hives over time. We conducted a prospective study of a large scale migratory bee keeping operation using high-frequency sampling paired with comprehensive molecular detection methods, including a custom microarray, qPCR, and ultra deep sequencing. We established seasonal incidence and abundance of known viruses, *Nosema sp.*, *Crithidia mellificae*, and bacteria. Ultra deep sequence analysis further identified four novel RNA viruses, two of which were the most abundant observed components of the honey bee microbiome (∼10^11^ viruses per honey bee). Our results demonstrate episodic viral incidence and distinct pathogen patterns between summer and winter time-points. Peak infection of common honey bee viruses and *Nosema* occurred in the summer, whereas levels of the trypanosomatid *Crithidia mellificae* and Lake Sinai virus 2, a novel virus, peaked in January.

## Introduction

Western honey bees (*Apis mellifera*) are highly social insects that live in colonies of ∼30,000 individuals [1], [2]. Honey bees are essential pollinators of agriculturally important crops including apples, almonds, alfalfa, and citrus. Current agricultural practices, such as large-scale monocultures, demand a seasonal abundance of honey bees in geographic locations incapable of maintaining sufficient pollinator populations year-round. Migratory beekeeping operations fulfill this need. For example, each February in the Central Valley of California 1.3 million honey bee colonies (∼50% of the U.S. honey bee population) are required for almond pollination [3], [4], [5]. Pollination of this and other U.S. crops is valued at ∼$15 billion annually [5].

There are numerous threats facing honey bee populations and the recent losses of honey bee colonies in the United States, Canada, and Europe is alarming. In the U.S., annual honey bee colony losses increased from 17–20% to 32% during the winter of 2006/07 with some operations losing 90% of their hives [6]. Average annual losses have remained high, averaging 32.6% from 2007–2010 [6], [7], [8]. One factor contributing to increased losses is Colony Collapse Disorder (CCD), an unexplained loss of honey bee colonies fitting a defined set of criteria [9], [10]. While factors such as pesticide exposure, transportation stress, genetic diversity, and nutrition affect colony health, the most significant CCD-associated variable characterized to date is increased pathogen incidence [10]. Although greater pathogen incidence correlates with CCD, the cause is unknown in part due to insufficient knowledge of the pathogenic and commensal organisms associated with honey bees [10], [11].

Parasitic threats to honey bee colonies include viruses, *Nosema*, bacteria, and *Crithidia*. The majority of honey bee infecting viruses are positive-sense single-stranded RNA viruses of the *Picornavirales* order. They include acute bee paralysis virus (ABPV) [12], black queen cell virus (BQCV) [13], Israeli acute bee paralysis virus (IAPV) [14], Kashmir bee virus (KBV) [15], deformed wing virus (DWV) [16], sacbrood virus (SBV) [17], and chronic bee paralysis virus (CBPV) [18] (reviewed in Chen and Siede, 2007 [19]). Several DNA viruses that infect honey bees have also been described [19]. Viral infections in bees can remain asymptomatic, or cause deformities, paralysis and/or death [19], [20]. Symptoms associated with specific viruses include wing deformities (DWV), hairless, dark, shiny bees (CBPV), swollen yellow larva and/or dark-brown larva carcasses in the cells of worker-bees (SBV) or queen-bees (BQCV), however accurate diagnosis requires molecular biology techniques as asymptomatic bees frequently test positive for one or more viruses [19], [21], [22], [23]. In addition to viral infections, honey bees are also readily parasitized by the microsporidia *Nosema* [19], [24]. Historically U.S. honey bees were predominantly infected by *Nosema apis*, but recently *Nosema ceranae* infections dominate [24], [25]. The effects of *Nosema* infection on individual bee and colony health are unclear [24], [26]. Some reports suggest infections decrease longevity and may lead to collapse [27], [28], [29], but since *Nosema* is widespread and often detected in healthy colonies its role in colony health requires further investigation [10], [26], [30]. Another fungal pathogen *Ascophaera apis*, the causative agent of Chalkbrood disease, kills infected larvae, but does not typically cause colony loss [31], [32]. Bacterial pathogens of honey bees include *Paenibacillus larvae* and *Melissococcus plutonius*, the causative agents of American and European Foulbrood disease [33], [34], [35], [36]. In addition to microbial infections, mite infestation (*Ararapis woodi*, *Tropilaelaps sp.*, and *Varroa destructor*) also weakens and kills honey bee colonies [37], [38]. Introduction of *V. destructor* mites, which feed on the hemolymph of developing honey bees and transmit viruses (DWV, KBV, IAPV), in the late 1980s was devastating to the U.S. honey bee population [39], [40], [41]. Notably, the restricted genetic diversity of the U.S. honey bee population may make it particularly susceptible to catastrophic and episodic losses [42], [43].

To gain a more complete understanding of the spectrum of infectious agents and potential threats found in commercially managed migratory honey bee colonies, we conducted a 10-month prospective investigation. Our broad-scale analysis incorporated a suite of molecular tools (custom microarray, polymerase chain reaction (PCR), quantitative PCR (qPCR) and deep sequencing) enabling rapid detection of the presence (or absence) of all previously identified honey bee pathogens as well as facilitating the detection of novel pathogens. This study provides a comprehensive temporal characterization of honey bee pathogens and offers a baseline for understanding current and emerging threats to this critical component of U.S. agriculture.

## Results

**Figure 1:**
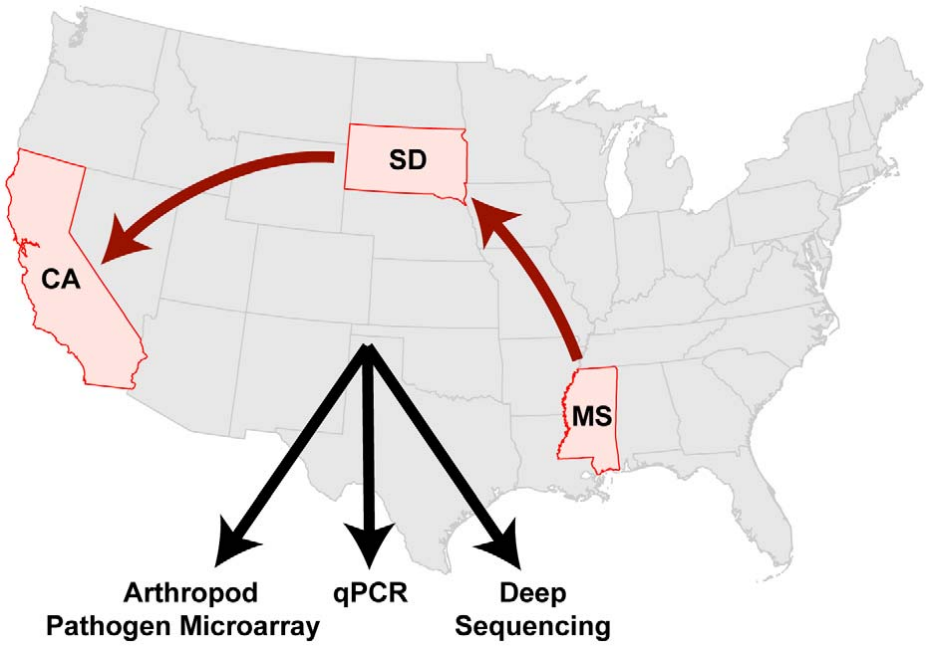
Temporal monitoring of the honey bee microbiome from 20 monitor colonies within a large-scale migratory U.S. beekeeping operation using a custom arthropod pathogen microarray, PCR, quantitative PCR, and ultra deep sequencing. The colonies were established with new queens in Mississippi (MS) in April 2009, moved to South Dakota (SD) in May 2009, and finally to California (CA) in November 2009; monitoring concluded in January 2010.

Following devastating losses suffered by U.S. commercial beekeeping operations in 2006–2007, we initiated a prospective study monitoring a typically managed, large-scale (>70,000 hives), migratory commercial beekeeping operation over 10-months. Honey bees from 20 colonies were consistently sampled beginning with the introduction of a new queen in April 2009 (Mississippi (MS), through transport to summer foraging grounds in South Dakota (SD), and transfer to California (CA) for almond pollination (Figure 1). During our study, these colonies were exposed to antimicrobial treatments, transportation stress, different pollen and nectar sources, and three distinct geographic locations: MS, SD, and CA, (U.S.A.).

A molecular analysis pipeline consisting of custom microarray, polymerase chain reaction (PCR), quantitative PCR (qPCR) and ultra deep sequencing was employed to characterize the honey bee microbial flora. Pathogen screening was performed using the “Arthropod Pathogen Microarray” (APM) built on the same design principles used for human pathogen microarray screening [44], [45]. The array's design couples highly-conserved nucleic acid targets with hybridization-based detection to identify previously uncharacterized organisms [46], [47], [48], [49], [50], [51]. Specifically, the APM was designed to detect virtually all known microbial parasites of insects. Endpoint PCR provided sensitive detection while qPCR documented abundance of select pathogens. Ultra deep sequencing facilitated the discovery of novel and highly divergent microbes. Together the results from our monitoring study provide insight regarding the incidence of virus and microbe infections in honey bee colonies.

### Arthropod Pathogen Microarray design and validation

The Arthropod Pathogen Microarray (APM) is a custom DNA microarray capable of detecting over 200 arthropod associated viruses, microbes, and metazoans. This DNA microarray includes oligonucleotides representing every arthropod-infecting virus with published nucleic acid sequence in the International Committee on Taxonomy of Viruses database as of November 2008 [52], [53]. Design principles used for APM oligonucleotides (70-mers) were based on previous pan-viral microarrays using ArrayOligoSelector (AOS) [54]. In addition, non-viral pathogens including, *Nosema* (microsporidia), *Crithidia* (trypanosomatid), *Varroa* (mite), *Tropilaelaps* (mite) and *Acarapis* (tracheal mite) as well as *Paenibacillus larvae* and *Melissococcus plutonius* bacterial species [53] were represented on the microarray (Table 1). This new diagnostic tool is composed of 1536 oligonucleotides, including viral, non-viral and positive control targets (Table 1). Array analysis is performed computationally using e-predict [54], [55]. The sensitivity of the APM was estimated to be 1.9×10^5^ viral genome copies (1 pg *Drosophila* C virus *in vitro* transcribed genomic RNA) in an *A. mellifera* RNA (1 µg) background (see Materials and Methods). Array specificity was confirmed by performing pathogen-specific PCRs in conjunction with nucleic acid sequencing. Test samples included honey bees from managed and feral colonies, *Vespula* sp. (yellow jackets), and *Bombus* sp. (bumble bees) (Table S1). A sample from a collapsed colony in Montana tested positive for the highest number of viruses (BQCV, DWV, KBV, IAPV) and documented the array's ability to simultaneously detect multiple pathogens. Analysis of symptomatic honey bees, such as hairless, shiny bees and bees with deformed wings, confirmed the presence CBPV and DWV, respectively [56], [57]. Likewise, analysis of *Varroa destructor* RNA validated the array's ability to detect mites and their associated viruses (DWV). Interestingly, pathogens normally associated with honey bees, DWV and ABPV, were also detected in a yellow jacket sample (*Vespula sp.*) obtained near a hive entrance from which the honey bees also tested positive for ABPV and DWV. We used the APM to detect several pathogens (BQCV, DWV, SBV and *Nosema*) in CCD-affected colony samples from an Oklahoma based migratory beekeeping operation (Feb. 2009). In total we detected and sequence confirmed ten previously characterized honey bee pathogens using the array including: CBPV, IAPV, DWV, ABPV, BQCV, SBV, KBV, *Nosema apis*, *N. ceranae* and *Varroa destructor*.

**Table 1:** Oligonucleotide targets for the Arthropod Pathogen Microarray.

**Dicistrovirus**	**Total: 264**
acute bee paralysis virus	38
black queen cell virus	42
Israeli acute paralysis virus	26
Kashmir bee virus	42
other Dicistroviruses	116
**Iflavirus**	**Total: 128**
deformed wing virus	22
honey bee slow paralysis virus	24
sacbrood virus	22
other Iflaviruses	60
**Other Virus Families**	**Total: 794**
Ascovirus	80
Baculovirus	138
Birnavirus	12
Cypovirus	98
Densovirus	110
Idnoreovirus	10
Iridovirus	46
Luteovirus	10
Nimavirus	20
Nodavirus	68
Okavirus	10
Poxvirus	74
Rhabdovirus	10
Tetravirus	30
Totivirus	10
**Unassigned Virus Families**	**Total: 88**
chronic bee paralysis virus	26
*Solenopsis Invicta* virus II	26
Acyrthospihon Pisum virus	12
Nora virus	12
kelp fly virus	12
**Bacteria**	**Total: 70**
*Achromobacter*	14
*Paenibacillus*	22
*Melissococcus*	10
*Enterococcus*	12
*Wolbachia*	6
*Brevibaccilus*	6
**Fungi/Protists**	**Total: 102**
*Crithidia*	20
*Nosema*	20
*Ascophaera*	10
*Aspergillus*	20
*Metarhizium*	20
*Hirsutella*	12
**Mites**	**Total: 80**
*Varroa*	32
*Tropilaelaps*	16
*Acarapis*	2
Nematodes	30
**Positive Controls**	**Total: 32**

### Temporal monitoring of 20 migratory honey bee colonies

Honey bee samples were collected during their travels from Mississippi through South Dakota to California resulting in a prospectively collected 10-month time-course of 431 data points, each consisting of 50–100 bees isolated separately from both the entrance (older foragers) and brood comb (younger house bees). Hives ^#^10, ^#^14 and ^#^19 were lost in December due to queen death or infertility. We analyzed all the entrance samples (5 bees per colony each time-point) using the APM.

#### *Nosema*

**Figure 2:**
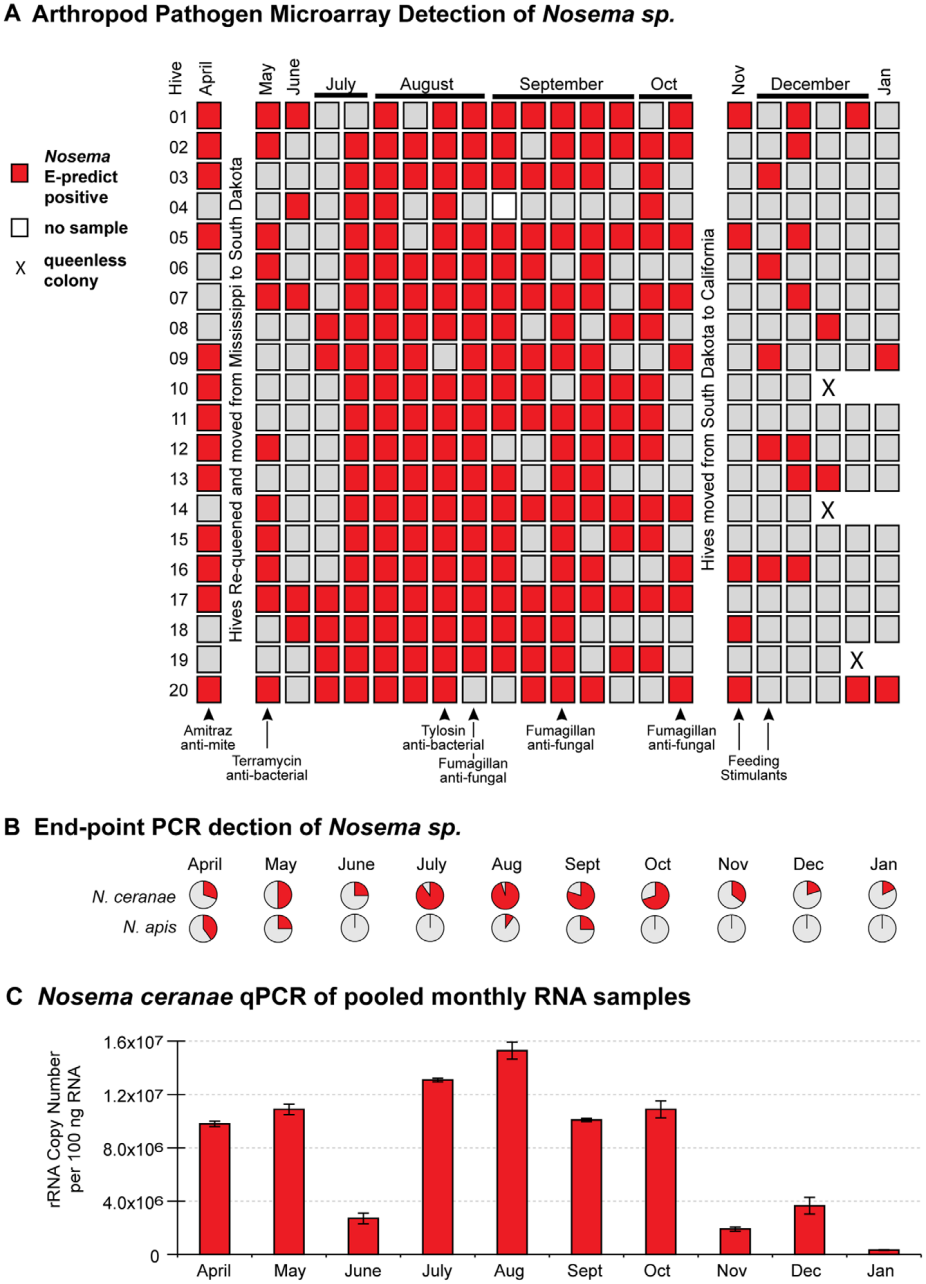
*Nosema* detection and quantification in time-course samples from 20 honey bee colonies. (A) Arthropod pathogen microarray detection of *Nosema sp.* in each colony (5 bees per sample) throughout the 10-month time-course. Colonies were managed using standard commercial beekeeping practices and treatments, which are listed below panel A and further described in Materials and Methods. (B) *Nosema ceranae* and *Nosema apis* incidence assessed by species-specific end-point PCR from a single time-point (n = 20) each month; the positive sample percentages in each pie-chart are indicated in red. (C) Relative abundance of *Nosema ceranae* throughout the time-course assessed by qPCR of pooled monthly RNA samples; quantification of rRNA copy number based on a standard curve as described in Materials and Methods.

There was an abundance of *Nosema* infections in our monitor colonies throughout the entire time-course. APM monitoring revealed that approximately half of the colonies in April and May were *Nosema* positive (Figure 2A). Notably, nearly every colony was infected during a surge in August and September. In order to determine which *Nosema* species was responsible for infections, each hive was analyzed at a single time-point per month by species specific PCR. In April and May, *N.apis* was predominant whereas in June, July, and October through December, *N. ceranae* was exclusively detected (Figure 2B). During the highest incidence of *Nosema* (August–September), 75% of all colonies were infected with *Nosema ceranae* and less than 25% with *Nosema apis*, most of which were co-infected with *N. ceranae*. Quantitative-PCR data from pooled monthly samples confirmed that *Nosema ceranae* was prevalent throughout the time-course and peaked in August (Figure 2C). While seasonal variation may play a role, an anti-fungal (Fumagillan) was used to abrogate *Nosema* infection [58] and may be responsible for the observed decrease in *Nosema* abundance from November to January (Figure 2).

#### Viruses

**Figure 3:**
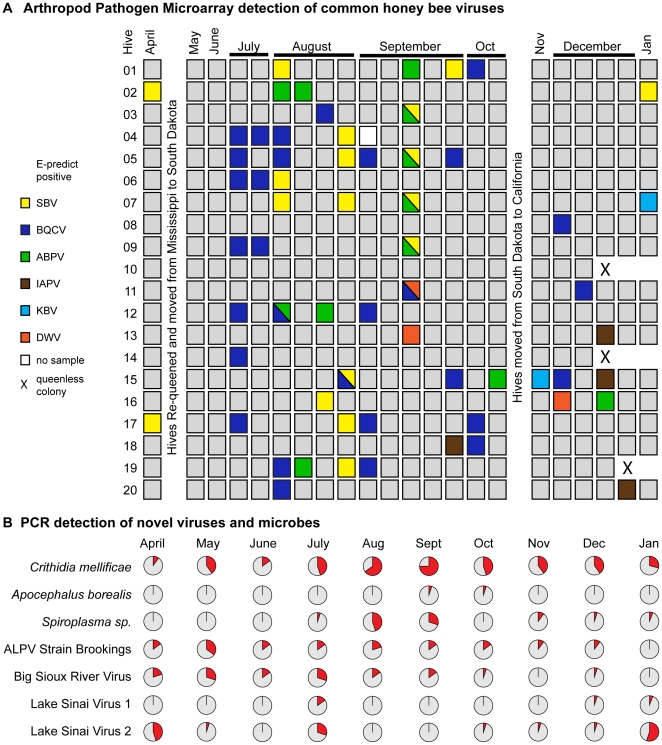
Detection of viruses and microbes in time-course samples from 20 honey bee colonies. (A) Arthropod pathogen microarray detection of viruses: sacbrood virus (SBV), black queen cell virus (BQCV), acute bee paralysis virus (ABPV), Israeli acute bee paralysis virus (IAPV), Kashmir bee virus (KBV), deformed virus (DWV) in each colony (5 bees per sample). (B) Incidence of select parasites assessed by end-point PCR from a single time-point each month (each chart n = 20, except January n = 17); the positive sample percentages in each pie-chart are indicated in red.

**Figure 4:**
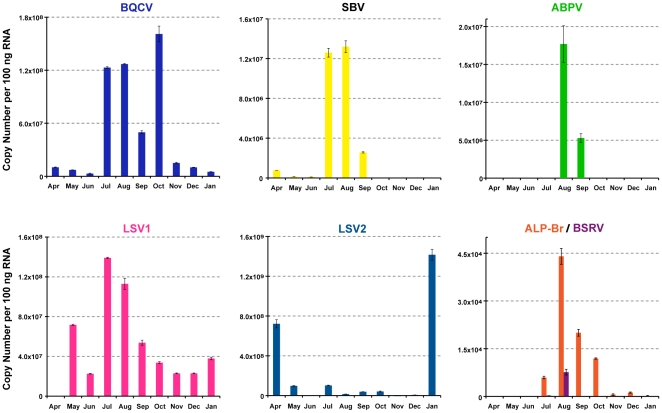
Relative abundance of select viruses assessed by RT-qPCR of pooled monthly time-course samples. Viral genome copy numbers per 100 ng RNA were calculated based on standard curves [(black queen cell virus (BQCV), sacbrood virus (SBV), acute bee paralysis virus (ABPV), Lake Sinai virus strain 1 (LSV1), Lake Sinai virus strain 2 (LSV2), aphid lethal paralysis virus strain Brookings (ALP-Br), and Big Sioux River virus (BSRV)]; multiplying reported values by 500 provides a copy number per bee estimate, as further described in Materials and Methods. LSV2, a novel virus, reached the highest copy number observed in this study in January 2010 (1.42×10^9^ copies per 100 ng of RNA sample; approximately 7.1×10^11^ copies per bee); note the y-axes on each graph are independently scaled.

The APM readily detected common honey bee viruses in samples collected throughout the time-course. In total, we report 69 virus incidences in 63 of 431 total samples (Figure 3). Overall virus incidence was sporadic, which we attribute to cycles of acute infection in predominantly healthy monitor colonies. The majority of infections occurred during July, August, and September when the monitor colonies were in South Dakota. The most prevalent virus infections observed during our 10-month study were SBV, BQCV and ABPV; however the frequencies of specific viruses were insufficient for statistical tests regarding pathogen association (see Materials and Methods). Other viruses including DWV, IAPV, and KBV were infrequently detected in the latter half of our time-course. A total of six double virus infections were observed, frequently involving ABPV or SBV. There were only two cases in which the same virus (BQCV) was detected in consecutive time points from a particular monitor colony (Figure 3A Hives #4 and #6). Typically a single virus was detected in multiple colonies at a given time-point and these infections did not persist. For example, there were waves of SBV infection in April and January and of BQCV in July and early August (Figure 3A). qPCR analysis of pooled monthly samples confirmed and extended APM findings. BQCV, SBV and ABPV levels peaked in mid-summer to early fall at 6.6×10^9^–8×10^10^ genome copies per bee (Figure 4 and Figure S1), consistent with previously characterized levels of these viruses [57], [59], [60].

#### Ultra deep sequencing, discovery of novel viruses

A summer South Dakota time-point (August 5, 2009) was selected for deep sequencing due to high *Nosema* load and the presence of several common honey bee viruses, including ABPV, BQCV and SBV. All expected microbes (*Nosema ceranae*, *Crithidia mellificae*) and viruses were detected [ABPV (39,352 reads aligned by BlastN e-value<1×10^−7^), BQCV (2,868 reads) and SBV (4,414 reads)]. In addition, we detected *Spiroplasma* sequences (70,407 reads) consistent with the presence of both *Spiroplasma apis* and *S. melliferum* (66 reads and 44 reads aligning to the RNA PolB gene of each, respectively).

Four distinct novel viruses were discovered via deep sequencing. Paired-end sequencing reads (2×63 nt) of unknown origin were screened by tBlastx [61] against all known insect viruses present in GenBank [62]. Screening hits with an e-value greater than 1×10^−3^ were used to target de novo contig assembly using the complete data set. Short contigs were screened by tBlastx against the non-redundant nucleotide database (NR) at an e-value threshold of 1×10^−5^. Hits to viral sequence, but not host sequences, were further assembled (see Materials and Methods). In each case, PCR primers were initially designed to bridge or confirm assembled contigs by Sanger sequencing. Confirmed contigs were extended with the PRICE assembler package (see Materials and Methods). In total, sequences from four novel viruses were recovered and Sanger validated. These include two members of *Dicistroviridae*, and two RNA viruses distantly related to *Nodaviridae*.

#### Aphid Lethal Paralysis virus strain Brookings

Investigation of contigs aligning to the Aphid Lethal Paralysis Virus genome, in the family *Dicistroviridae*, recovered a 4,125 nt contig (GenBank Q871932) spanning the RNA-dependent RNA Polymerase (RdRp) gene, the internal ribosome entry site (IRES) structure and the capsid coding region. The recovered sequence aligned with 83% nucleotide and 89% amino acid identity to the canonical ALPV genome over the RdRp gene. The two viruses shared 97% nucleotide homology along 171 nt of the IRES. The high sequence similarity between this new isolate and canonical ALPV makes it unclear whether this is a novel species or a new strain of ALPV. Regardless, ALPV has not previously been reported in association with honey bees. We propose the designation ALPV strain Brookings (after the SD county from which the virus was isolated). Specific PCR primers were designed for the Brookings strain and used to analyze additional time-course samples, resulting in detections on thirty distinct occasions, including in Mississippi, South Dakota and California. Incidence peaked in May, when 7 out of 20 hives were infected, whereas maximum abundance occurred in August albeit at a relatively low level, 4.42×10^4^ copies per 100 ng of RNA sample (approximately 2.21×10^7^ copies per bee), as compared to previously characterized honey bee viruses (Figures 3 and 4). Frequent detection of ALPV strain Brookings throughout the time-course from multiple geographic locations suggests that this virus is not simply a “passenger” obtained from forage (nectar and pollen) shared with other insects. However, further investigation is required to determine whether ALPV strain Brookings is a honey bee pathogen.

#### Big Sioux River virus

A second novel dicistrovirus, designated Big Sioux River Virus (BSRV) after its place of discovery, is most similar to the *Rhopalosiphum padi Virus* (RhPV). Four contigs of size 1473, 861, 1164 and 1311 nt (GenBank JF423195-8) derived from the non-structural region, the IRES, and the capsid gene. BSRV shares low amino acid identity with RhPV; only 78% in the non-structural region and 69% in the capsid gene. This level of amino acid divergence is consistent with the taxonomic rank of a new species (Figure S2). Twenty-eight incidences of BSRV were detected from 197 time-course samples by specific PCR with most individual colony detections occurring in samples collected from April to July 2009 in Mississippi and South Dakota. Incidence was low from October onwards (Figure 3B). Peak abundance was 7.64×10^3^ copies per 100 ng of RNA sample (approximately 3.8×10^6^ copies per bee) and occurred in August (Figure 4). Of note, BSRV associated significantly with *Nosema apis* infections (p = 0.003, OR 6.0) and also with ALPV-Brookings (p = 0.014, odds ratio (OR) = 4.5).

#### Lake Sinai Virus strain 1 and 2

Three contigs had significant alignment to chronic bee paralysis virus (CBPV) and members of the family *Nodaviridae*. Both the individual reads and our initial contigs were further assembled and extended using the complete data set (see Materials and Methods). Two separate contig sequences (∼5.5 kb each) were generated by de novo assembly. Both contigs were confirmed by specific PCR and Sanger sequencing. The first contig represents a novel RNA virus that we designate Lake Sinai virus (LSV1) (HQ871931), after Lake Sinai in Brookings County, South Dakota. The second contig also represented a related, yet divergent (71% nt identity), RNA virus which we designated Lake Sinai virus 2 (LSV2) (HQ888865). The 5′ end of LSV1 was determined by RACE (rapid amplification of cDNA ends). The 5′ end of the LSV2 assembly was within 57 nt of the LSV1 RACE results [18], [57]. The 3′ ends of both viruses were refractory to traditional RACE methods and attempts at 5′ RACE on the negative strand were also unsuccessful. Denaturing gel electrophoresis coupled with Northern bot analysis using three distinct LSV-specific probes indicated that only the monopartite genome and no subgenomic RNA species were present (Figure S4).

**Figure 5:**
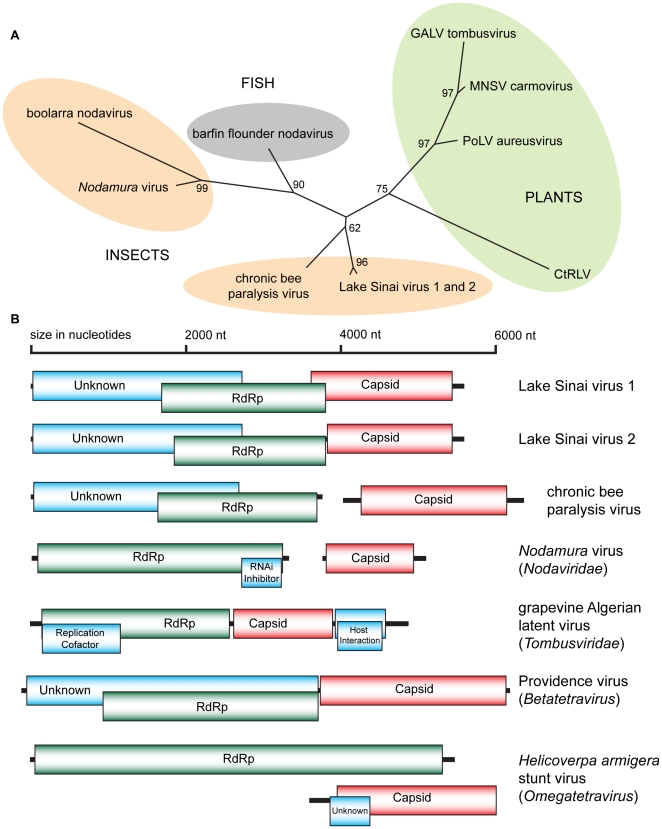
Phylogenetic placement and genome organization of Lake Sinai viruses. (A) RdRp amino acid phylogeny of the *Nodavirales* superfamily. Lake Sinai virus strain 1 (LSV1; HQ871931), Lake Sinai virus strain 2 (LSV2: HQ888865), chronic bee paralysis virus (CBPV; NC010711), boolarra virus (BoV; NC004142), *Nodamura* virus (NoV; NC002690), barfin flounder nodavirus BF93Hok (BFV; NC011063), grapevine Algerian latent virus (GALV; NC011535), melon necrotic spot virus (MNSV; NC001504), pothos latent virus (PoLV; NC000939) and carrot red leaf virus (CtRLV; NC006265). Protein sequences were aligned by ClustalW and a tree generated by the Neighbor-Joining method with 100 replicates [102] (B) Genome organization of the Lake Sinai viruses and similar RNA viruses.

Both LSV genomes display similarities to the RNA1 molecule of chronic bee paralysis virus (CBPV) with predicted open reading frames (ORFs) of similar size and arrangement with the notable exception that LSV1 and 2 ORFs are contained on a single RNA rather than in the bipartite configuration of CBPV [18], [57] (Figures 5B and Figure S4). LSV1 and 2 possess the Orf1 gene, which is of unknown function, with predicted products (of 847 and 846 aa) previously unique to CBPV (853 aa). The Orf1 genes of LSV1 and CBPV share minimal (18%) amino acid identity. All three viruses encode an RdRp that partially overlaps and exists in a frame shift with respect to Orf1 [18]. Both LSVs possess a triple stop codon within 10 residues of the end of the Orf1 gene whereas CBPV has two adjacent stop codons. The RdRp genes are considerably more conserved with 80% identity between the two LSV strains and 25% amino acid identity between them and CBPV. Both LSV RdRp genes have the DxSRFD and SG amino acid motifs in the NTP binding pocket (residues 375–380 and 436–437 in LSV1) conserved between the families *Nodaviridae*,*Tombusviridae* and CBPV. An amino acid phylogeny of the *Nodavirales* superfamily RdRp places the LSV strains on the same branch as CBPV, and separated from the larger *Nodavirus* and *Tombusvirus* families (Figure 5A).

As previously noted, the capsid protein of LSV1 and 2 is encoded on the same RNA as Orf1 and the RdRp unlike that of CBPV, which possesses a bipartite genome (Figure 5B). The capsids of LSV1 and 2 have significant profile similarity to the capsid gene of *Nudaurelia capensis beta-tetravirus* by HHpred [63] (e-value 1.0×10^−26^) and they exhibit weak direct protein alignment by Blastx (e-value 1.0×10^−04^). Similarity to *tetravirus* capsid genes consistently outranked similarity to CBPV or *nodavirus* capsids by these methods. *Tetraviruses* are not close relatives of the *Nodavirales* superfamily, although *Betatetraviruses* have a similar monopartite genome organization to LSV (Figure 5B). LSV1 and 2 share 70% amino acid identity over the capsid. The LSV1 capsid overlaps the RdRp gene in the +1 reading frame for 125 nt before ending in a pair of stop codons (separated by two residues). The LSV2 capsid is in frame with the RdRp and separated by 18 nt without a redundant stop codon.

Seven of twenty hives sampled on August 5, 2009 were positive for LSV1 and an additional five hives in the time-course, from July (SD) and January/February (CA) were found to be positive for LSV1, all with greater than 95% nucleotide identity. LSV2 was more prevalent and was detected by PCR in 30 of 197 time-course samples from all three geographic regions. LSV2 incidence surged in April, July and January during which over a third of all 20 monitor hives were infected. Strain specific qPCR demonstrated high abundance (≥2×10^6^ copies per 100 ng RNA) of both LSV strains in our monitor colonies throughout the majority of the time-course (Figure 4). LSV1 copy number peaked in July, at 1.39×10^8^ copies per 100 ng of RNA sample (approximately 7.0×10^10^ copies per bee). Notably, LSV2 was the most abundant virus detected in this study (∼10^11^ copies per bee). Copy number peaked in both April and January, at 7.22×10^8^ copies per 100 ng of RNA sample (approximately 3.61×10^11^ copies per bee) and 1.42×10^9^ copies per 100 ng of RNA sample (approximately 7.1×10^11^ copies per bee), respectively.

Positive sense RNA viruses, like LSV 1 and 2, utilize a negative strand template to produce viral genome copies, therefore detection of the negative-strand intermediate is indicative of an actively replicating infectious virus [39], [64], [65]. We used negative-strand specific RT-PCR to detect the replicative forms of both LSV1 and LSV2 (Figure S5). cDNA synthesis reactions were performed using tagged negative strand-specific LSV1 and 2 primers followed by exonuclease I digestion of excess unincorporated RT-primers [65] (Materials and Methods and Table S2). PCR amplification using a tag-specific forward primer and LSV-specific reverse primers confirmed the presence of the replicative forms of both LSV1 and LSV2 in the July RNA sample (Figure S5). Together, this data and the abundance of LSV1 and 2, compared to other significant honey bee viruses, suggests that LSV1 and LSV2 are novel honey bee viruses that may play significant roles in colony health.

#### *Crithidia mellificae*

**Figure 6:**
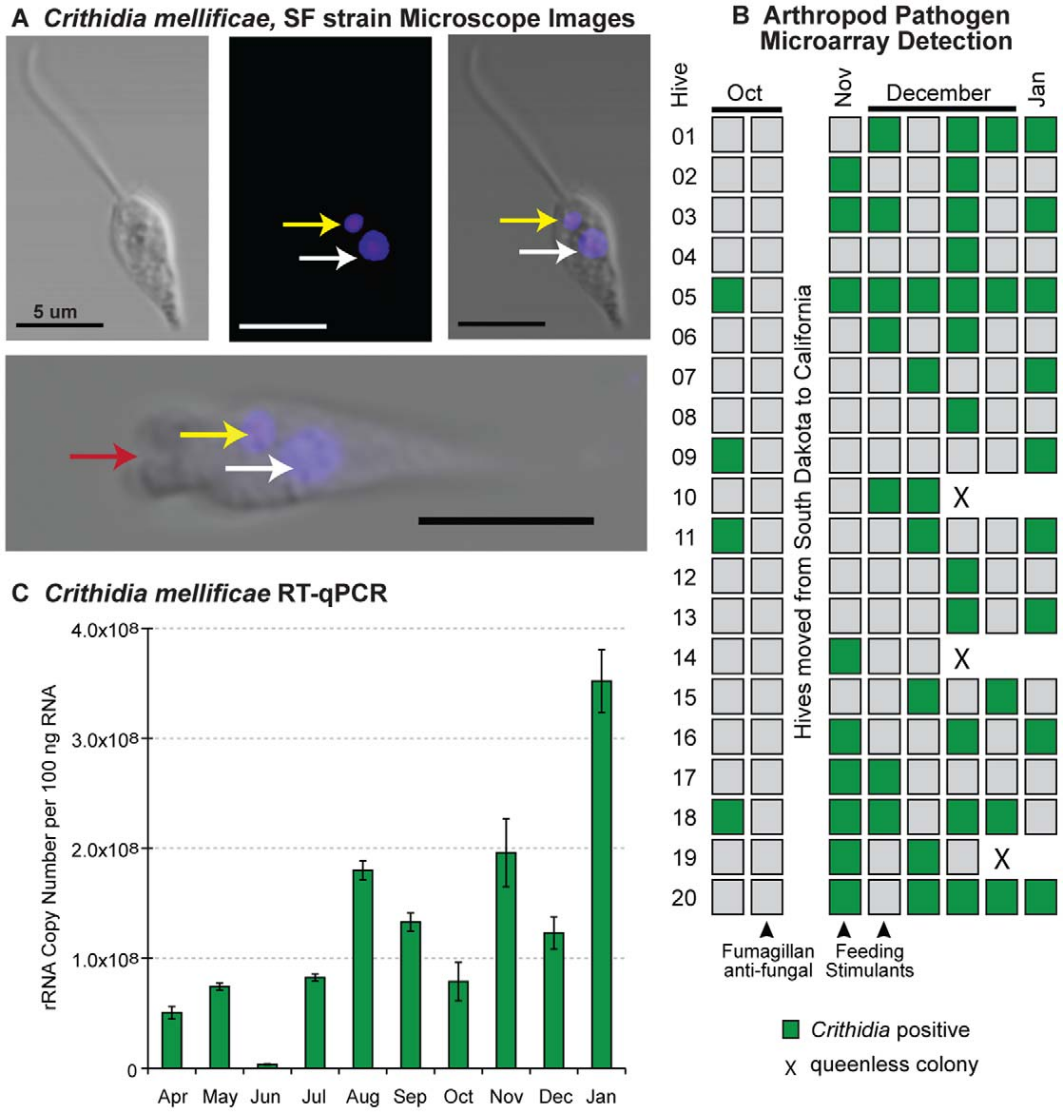
*Crithidia mellificae*, SF strain detection and quantification. (A) Light and fluorescent microscope images illustrate key features of this trypanosomatid parasite including DAPI stained kinetoplast DNA (yellow arrow) and nuclear DNA (white arrow), as well as the flagellar pocket (bottom panel, red arrow); scale bar = 5 µm. (B) Arthropod pathogen microarray detection of *Crithidia mellificae* in each colony (5 bees per sample) from October 2009 to January 2010. (C) Relative abundance of *Crithidia mellificae* throughout the time-course as assessed by RT-qPCR of pooled monthly time-course samples; quantification of rRNA copy number based on a standard curve as described in Materials and Methods.

The broad scope of our microarray platform enabled identification of an unexpected microbe, *Crithidia mellificae*, in our time-course samples (Figure 6). Given that *Crithidia bombi* is a bumble bee pathogen and trypanosomatids were previously described in honey bees [9], [66], [67], 5 unique oligonucleotides each from *Crithidia oncopelti* and *C. fasciculata* rRNA sequences were included on the microarray. Oligonucleotides from these two distantly related organisms were predicted to hybridize to all other *Crithidia* species with published sequence [53]. Three oligonucleotides and their reverse complements derived from *Crithidia oncopelti* were repeatedly detected in samples throughout the time-course. Pilot Sanger sequencing of randomly amplified genomic DNA from a honey bee intestinal sample yielded a 121 base-pair (bp) stretch of the kinetoplast minicircle with 74% homology to the *Crithidia fasciculata* kinetoplast (BlastN e-value = 3.5×10^−8^). Specific PCR retrieved 593 nt of the GAPDH gene to confirm phylogenetic placement [68].

We sought to further characterize this parasite by microscopy, PCR, culturing and DNA sequencing. Honey bee intestines were dissected in a sterile environment from which *Crithidia mellificae* was cultured. Light microscopy of these parasites enabled visualization of the flagella and motility (Figure 6; Figure S6 and Figure S7). Fixed sample imaging facilitated DAPI visualization of the kinetoplast DNA, as well as nuclear DNA (Figure 6). Previous studies describing trypanosomatids in honey bees lacked DNA-sequencing data with the exception of Cox-Foster *et al.* (2007) who published a 715-nt sequence of 18S ribosomal RNA that was too conserved between trypanosomatids for precise taxonomic assignment [9]. Together, the features observed by microscopy (flagella and kinetoplast) and phylogenetic analysis unambiguously identify this species taxonomically. We have deposited the GADPH sequence (JF423199) for future molecular identification, and genomic sequencing of *C. mellificae* is underway.

In order to specifically monitor *Crithidia mellificae*, additional oligonucleotides complementary to the *C. mellificae* rRNA and kinetoplast sequence were designed and included on the APM beginning in October 2009. These additional oligonucleotides enabled robust *C. mellificae* detection in later time-course samples, 33% of which tested positive (Figure 6). In addition, we screened samples throughout the time-course (April 2009–Jan. 2010) by PCR and qPCR specific to the *C. mellificae* rRNA gene. *C. mellificae* infection was detected by PCR at every time-point and in turn from every geographic location sampled in our study (MS, SD and CA). Likewise, *C. mellificae* was readily detected in pooled monthly RNA samples by qPCR throughout the year (Figure 6C). In contrast to BQCV, SBV, ABPV and *Nosema ceranae*, which exhibited peak levels in late summer and early fall, peak trypanosomatid levels occurred in January 2010. Despite this, *C. mellificae* infections statistically associated with *N. ceranae* infections (Chi Square p = 0.004, OR = 3.1). *C. mellificae* was also detected in numerous hobbyist and study hives in the San Francisco Bay Area (CA), as well as samples from a CCD-affected apiary in Oklahoma, indicating wide geographic distribution (Table S1).

#### *Spiroplasma melliferum* and *S. apis*

*Spiroplasma*, a close relative of the genus *Mycoplasma*, are bacterial parasites that have been implicated as pathogens of insects, vertebrates and plants. Strains of *spiroplasma* similar to flower-associated parasites were identified as a pathogen of honey bees in France, *Spiroplasma apis* [69], and the United States, *Spiroplasma melliferum* [70]. Pilot Sanger sequencing of a pooled honey bee sample (August 2009) identified an rRNA-derived sequence from a *Spiroplasma*. Pan-*spiroplasma* and pan-*mycoplasma* PCRs targeting the 16S rRNA gene detected sporadic infections over most of the time-points and a surge of 9 infections in August and 6 infections in September [71]. Sequence data indicates that these isolates have high homology to previously identified *spiroplasma* isolates (>98% nucleotide identity). *Spiroplasma* infections had strong associations with *N. ceranae* (Chi Square p = 0.015, OR = 7.2) and *C. mellificae* (p = 0.000076, OR = 16.3), however this may be an artifact of the short surge of *Spiroplasma* coinciding with a period of high *Nosema* load.

#### Phorid fly (*Apocephalus borealis*)

*Apocephalus borealis*, phorid flies, have previously been associated with bumble bee parasitism [72] and have recently been described as a parasite of honey bees in the San Francisco Bay Area [73]. *Phoridae* family members (*e.g. Pseudacteon* sp.) are well-characterized parasites of ants and other insects. These flies lay eggs inside the insect hosts, which are in turn consumed by the larvae during development. Although, *A. borealis* parasitism of honey bees is uncommon, we analyzed our time-course samples for the presence of phorid rRNA by PCR. Pooled monthly samples were weakly positive for *Apocephalus borealis* in December and January (Figure S3) and two individual hive samples produced robust amplicons. We sequenced PCR amplicons from two individual (September 2009 Hive ^#^7 and October 2009 Hive ^#^10) and one pooled-monthly (December 2009) samples and determined that the phorid rRNA sequences from our time-course shared 99% similarity to honey bee-parasitizing phorids captured in San Francisco. This is the first report of phorid flies in honey bee samples outside of California and thus expands their known geographic range (SD, CA), although the range *A. borealis* as a bumble bee pathogen extends across North America [74].

## Discussion

The importance of honey bees to global agriculture and the emergence of CCD calls for increased longitudinal monitoring of infectious processes within honey bee colonies. The data presented herein represent the finest resolution time-course of honey bee associated microbes to date. We demonstrate the utility of an arthropod pathogen microarray (APM) for simultaneous detection of numerous pathogens and the power of ultra deep sequencing for viral discovery. Several previous studies examined honey bee samples from diseased or CCD-affected and healthy colonies [9], [10], [20], [75], [76], but few have temporally monitored multiple pathogens [60], [77], [78]. Although these studies differed in sampling strategy, geography, colony management (*e.g.* migratory commercial versus stationary hobbyist, chemically treated versus organic), and pathogen monitoring technology (*e.g.* serology, PCR, spore counts, microarray) they provide a framework for our surveillance of previously characterized honey bee pathogens.

*Nosem*a infection was prevalent in our 20 monitor colonies. *N. ceranae* was the predominant species. *N. apis* was detected in individual colony samples in April (Mississippi) and May (South Dakota), but was undetectable in pooled monthly samples, indicating relatively low levels. *N. ceranae* abundance peaked in early-spring and late-summer. Lower *N. ceranae* levels from November to January likely reflects antifungal (Fumagillan) treatments applied in the fall, but may also represent natural seasonal variation [58]. In comparison, another U.S.-based (Mississippi, Arkansas) study, which calculated *Nosema* levels using qPCR of genomic DNA calibrated to spore counts, also reported overall dominance by *N. ceranae*, but higher *Nosema* levels in November 2008 as compared to March 2009 [79]. *Nosema* spore count data from non-CCD and CCD-affected colonies in California and Florida was not significantly different and approximately 50% of the colonies assayed were infected [10]. Data from European studies indicate varying prevalence of *N. apis* and *N. ceranae* [25], [29], [80], [81]. For example, a retrospective analysis of honey bee samples from Spain, Switzerland, France and Germany indicated peak levels of *Nosema* (presumably *N. apis*) in early spring and mid-winter from 1999 to 2002, whereas from 2003 to 2005 *Nosema* incidences remained relatively high throughout the year, a result the authors attribute to increased prominence of *N. ceranae* associated with recent increased bee losses [29]. In contrast, a recent (2005–2009) time-course study in Germany demonstrated greater *Nosema* incidence in the spring, detected *N. apis* more frequently than *N. ceranae*, and found no correlation between colony loss and *Nosema* infection [80]. Variable *Nosema species* prevalence and abundance at both the apiary and individual colony level indicate that standardized, molecular biology-based monitoring of large sample cohorts is required in order to understand the dynamics of *Nosema* infection, which are likely influenced by multiple factors including host genetic variation, climate, exposure levels, and treatment regimes [79], [82]. Recently, higher levels of *Nosema bombi* were detected in North American bumble bee species experiencing population decline [83]. Although, like CCD, the causes of bumble bee decline are complex and not fully characterized, this report underscores the importance of further characterizing the epidemiology and pathogenicity of *Nosema*.

We monitored the incidence of all known honey bee viruses, discovered 4 new honey bee associated viruses, and quantified the relative abundance of select viruses in time-course samples. Overall, no chronic infections of previously characterized honey bee viruses were observed and our data suggest that healthy colonies are undergoing constant cycles of viral infection. The most prevalent, previously characterized viruses in our study were BQCV, ABPV and SBV. The peak incidence of BQCV (25%) occurred in July, whereas ABPV (6.3%) and SBV (12.5%) peaked in August. Summer peak virus incidence was also reported in a PCR based honey bee virus (BQCV, ABPV, and SBV) survey of 36 geographically distributed apiaries in France (BQCV, ABPV, DWV, SBV, CBPV, KBV) [78], a qPCR time-course study of 15 colonies in England (BQCV and ABPV) [60], and an unpublished East-coast U.S. based survey (BQCV) [19]. Another virus, invertebrate iridescent virus-6, claimed to be associated with CCD and prevalent (75%) in healthy colonies but not supported in subsequent analysis [84], [85], was never detected by the APM (n = 431), end-point PCR (n = 197), or in any of the 20 samples that were deep sequenced [86].

Seasonality of specific pathogens in our time-course study representing 2,155 individual bees from 431 samples varied, although many including BQCV, APBV, SBV, *Nosema*, exhibited reduced June and peak August levels. Peak incidences of these organisms in the spring and late summer are likely attributable to increased brood rearing [19], [78], [87] and foraging during these seasons [88]. Increased brood rearing during the summer, results in a greater number of bees capable of transmitting pathogens to other members of the colony living in very close proximity [19]. Honey bee viruses are transmitted vertically via infected queens and horizontally via the oral-fecal route or through the exoskeleton [19], [21]. Foraging activity also increases pathogen exposure [88] and may also stress the bees so that inapparent infections reach detectable levels. Although other sources of stress, such as transportation and poor nutrition, are hypothesized to increase pathogen levels [10], these factors were minimal during the summer when the monitor colonies were stably situated in South Dakota foraging on diverse pollen and nectar sources, including alfalfa (*Medicago sativa L.*), sweet clover (*Melilotus spp.*) and a variety of other flowering plants in June with increasing availability of corn (*Zea mays ssp.*) and soybean (*Glycine max*) pollen later in the summer. Notably, these colonies were part of a typically managed commercial beekeeping operation and therefore received nutritional supplements, protein paddies and sugar syrup throughout the year (Materials and Methods). Adequate monitor colony nutrition may have played an important role in the rapid virus clearance observed in our study. Although further experimental validation is needed, recent work examining the effects of nutrition on DWV titer in caged-bee studies demonstrated that viral titer was reduced by pollen and protein supplementation [89]. In addition, anti-mite and antimicrobial treatments in the spring and late-fall may have accounted for the lower pathogen levels at those times of year and in turn for the relatively high levels during the summer (Materials and Methods). We did not observe either increased incidence or abundance of any of the microbes and viruses monitored in our study after long distance transport.

Although several monitor colonies were lost (n = 3; one unfertile (drone laying) queen, two queen-less colonies) and many (n = 8) had fewer than 6 frames of bees in February 2010, none exhibited CCD characteristics and none of the numerous viruses and microbes we surveyed correlated with the weak colonies. Interestingly, our sample cohort had very few incidences of IAPV and DWV. IAPV, a virus that has received much attention due to its correlation with CCD-affected samples in an early study [9], although not in a subsequent expanded study [10], was detected in our monitor hives in December. The colonies in our study cleared or reduced IAPV infection to levels below detection within one week, indicative of a mild infection (Figure 3). IAPV infection has been shown to cause paralysis and death in mini-colony and cage studies [14], [90], although its role in CCD is unclear [10], [91], [92]. Likewise, DWV incidence in our time-course samples was very low (0.7%) and presumably cleared rapidly. In contrast, a French time-course documented increased DWV incidence throughout the year (spring 56%, summer 66%, autumn 85%) [78] and two U.S. studies also report high DWV incidence [19], [76]. Our results are not indicative of poor DWV detection by the array or our sampling strategy, since DWV was detected in both entrance and interior samples from other colonies. In addition, DWV-specific PCR of pooled monthly time-course samples was negative (Figure S3). Therefore negligible DWV in our monitor colonies may be attributed to low exposure and/or good colony health. A thorough one-year investigation of virus (ABPV, BQCV, DWV) and *V. destructor* in England found a correlation between DWV copy number and over-winter colony loss [60]. Lack of DWV in our monitor colonies is consistent with low *Varroa destructor* incidence, since mites are known to transmit DWV [39], [93], [94]. Low incidence of both DWV and *V. destructor* in our study may be partially attributed to our analysis of entrance samples, which consist of actively foraging and/or guarding adult bees. Since *Varroa* mites parasitize larva they are more readily detected in larva and young bee samples as well as hive bottom boards. More significantly, monitor colonies received miticide treatments in order to reduce *V. destructor* burden.

Deep sequencing analysis revealed the presence of four novel viruses (ALPV-Brookings, BSRV, LSV1 and LSV2), illustrating the power of deep short read sequencing and de novo assembly for virus discovery. Significantly, LSV2 was the most abundant single component of the honey bee microbiome in our study, and it is likely that the reason this virus has previous gone undetected is the fact that the Lake Sinai viruses are extremely divergent from all known insect viruses in both amino acid identity and genome organization. The non-structural genes of LSV are most closely related to CBPV, a known pathogen of honey bees [57], as well as other members of the *Nodavirales*. However, the capsid gene and monopartite genome structure resemble *tetraviruses* and together position this virus closer to species such as the Providence virus, which similarly has a monopartite genome, a *Nodavirales*-like polymerase and a *tetravirus*-like capsid. Since the presence of viral nucleic acid does not necessarily indicate infection, as pollen pellets of infected and non-infected workers are known to harbor honey bee viruses [88], we confirmed the presence of the replicative forms of LSV1 and 2 in time-course samples. The enormous magnitude of LSV throughout the time-course also suggests that these are bona fide honey bee viruses. LSV2 was the most abundant virus in our study and exhibited a unique seasonality. It is intriguing that peak LSV2 copy number per bee occurred in April (∼3.6×10^11^) and January (∼7.1×10^11^) since colonies typically collapse during the winter months. In contrast, LSV1 copy number peaked in July, similarly to the previously described honey bee viruses monitored in our study. Frequent detections of both ALPV-Brookings and BSRV (∼15% incidence in the time-course) by PCR screen in different geographic regions argues against simple carryover from other insects during foraging, but does not rule out potential re-infection from stored pollen (bee bread) [88]. Research to determine the potential pathogenicity of these four new viruses in honey bees is underway. There are a number of previously identified honey bee viruses described on the basis of serology and electron microscopy (Bee virus X [95], Bee virus Y [96] Arkansas bee virus [97] and Berkeley bee virus [98] for which no nucleic acid sequence information is available in public databases. Without such data we cannot preclude the possibility that these previously described viruses overlap with our novel viruses, however we were not able to gain access to any nucleic acid or serological reagent to address the question directly. Regardless, the nucleic acid sequences of the viruses reported herein are attached to publically accessible records in the form of GenBank accessions (LSV1 - HQ871931, LSV2 -HQ888865, ALPV Strain Brookings - Q871932 and BSRV - JF423195-8), such that any future viral samples may be directly compared, or if historical samples can be found and analyzed, they too can be compared.

*Crithidia mellificae* was readily detected throughout the time-course. In contrast to most other prevalent microbes and viruses, relative *Crithidia* levels peaked in the winter (January 2010). The effects of *C. mellificae* on the honey bee host remain relatively uncharacterized compared to those of *C. bombi* on bumble bee, which include reduced worker fitness and colony survival [66], [99]. To date, there are only a few reports of *C. mellificae* infection of honey bees in the literature including early work describing the first isolation and culture of this organism in 1967 from Australian honey bees [67]. This work tested the effect of feeding *C. mellificae* to honey bees and demonstrated similar mortality rates in infected and uninfected bees [67]. More recently, similar trypanosomatid prevalence and loads were reported in CCD-affected colonies and healthy controls [9], [10]. Although current data suggest that *C. mellificae* does not dramatically affect colony health additional pathogenesis research in honey bees is warranted considering the detrimental effects of *C. bombi* on bumble bee colonies.

The importance of honey bees in agriculture and the emergence of CCD underscores the need to monitor honey bee associated viruses and microbes in healthy colonies over time. The confinement of *Spiroplasma* infection to a two-month window demonstrates the value of time-course sampling as opposed to single-collection screens. The development of high throughput platforms, such as the APM, will facilitate monitoring of exogenous agents in order to better understand their effect on honey bee health and survival. Our discovery and genomic characterization of four new viruses will facilitate future monitoring. Temporal characterization of these and the other microbes described herein offers a more complete view of the possible microbe-microbe and microbe-environment interactions. Further studies examining any subtle or combinatorial effects of these novel microbes are required to understand their role in colony health. Increased analysis of prospectively collected samples is essential to address the hypothesis that either one or more viruses and/or microbes cause CCD. To our knowledge, this is the first U.S. honey bee pathogen monitoring study to report both comprehensive pathogen incidence and relative abundance of specific pathogens over time. Results from our molecular analysis pipeline (APM, PCR, qPCR, ultra deep sequencing) provide a basis for future epidemiologic studies aimed at determining the causes of CCD.

## Materials and Methods

### Collaborating commercial beekeeping operation information

Twenty monitor hives were established in April 2009 by a large-scale (>72,000 hives), migratory commercial beekeeping operation (Mississippi, California, and South Dakota, U.S.A.) that experienced CCD-losses in 2007/08. Standard beekeeping management practices for an operation of this size were employed. Treatment regimes throughout the year were as follows: (1) anti-mite treatment April 2009, just prior re-queening – amitraz; (2) antibacterial treatment May 2009 - oxytetracycline hydrochloride (OTC) (Terramycin™); (3) anti-fungal (*Nosema sp.*) treatment August 25, September 12, and October 13, 2009 - fumagillan; (4) antibacterial treatment late August, early September, 2009 - tylosin tartrate; (5) anti-mite treatment September 12, 2009, after harvesting honey; (6) anti-mite treatment – early November and early December 2009 - essential oils from lemon grass and spearmint (Honey-B-Healthy™). Honey bees colonies were periodically supplemented with sugar syrup and protein supplement. In April (1 gallon) and October (2 gallons) bees were fed 50% (weight/volume) sucrose; in November all colonies received 3 gallons of a 1∶1 mixture of high fructose corn syrup-55 (HFCS-55, 55% fructose, 42% glucose) and sucrose syrup. Additional sugar syrup was given to colonies based on colony weight (<80 lbs - 3 gallons, 80–90 lbs - 2 gallons., 90–100 lbs – none). This operation experienced an average 18% colony loss from November 2009 to February 2010. Colonies with younger queens (≤2 years old) experienced 11% loss, whereas colonies with older queens experience 21% loss.

### Honey Bee sampling and storage

Samples (∼50–100 bees) were collected into 50 mL Falcon tubes using a modified hand-held vacuum cleaner from both the entrance and interior of the hive and immediately put on dry ice for overnight shipment to our laboratory. Samples were stored at −80°C until RNA extraction; excess bees were archived for long-term −80°C storage. Time-course samples were collected monthly from April 15 (week 1) through July 14 (week 14), 2009 and weekly samples were attempted thereafter, however due to inclement weather or shipping logistics the samples for weeks 15, 28–30, 32, and 39–41 were not collected. A total of 864 samples were obtained and 431 exterior samples were analyzed.

### Honey bee sample preparation

We determined that analysis of five honey bees per sample was sufficient for our colony monitoring project. Arthropod pathogen microarray (APM) analysis of test samples revealed that combined analysis of 5 bees reproducibly detected most, if not all, of the pathogens detected from 10 or 15 independently analyzed bees from the same sample. In addition, we confirmed the consistency of APM results by performing multiple analyses of a single RNA sample. Based on our test results and practical sample handling considerations, we reasoned that repeated analysis of 5 bees from each colony over-time (115 bees per colony) was sufficient for this study.

Honey bee samples, 5 bees per colony each time-point, were homogenized in 1 mL 50% TRIzol Reagent (Sigma) and 50% phosphate buffered saline (PBS, UCSF Cell Culture) solution in a 2 mL micro-centrifuge tube containing one sterile zinc-coated steel ball bearing (5 mm) using a TissueLyzer II (Retsch), for 4 minutes at 30 Hz. RNA was isolated according to TRIzol Reagent (Invitrogen) manufacturer's instructions. In brief, TRIzol reagent honey bee homogenate was combined with 0.1 ml chloroform and mixed by vortexing for 5 seconds, samples were incubated at room temperature for 5 minutes, prior to centrifugation for 10 minutes at 13,200× g in a table top centrifuge. Next, 700 µL of the aqueous phase was transferred to a new microfuge tube containing 490 µL isopropanol. Following mixing, the samples were incubated at −20°C for 20 minutes and then either centrifuged (13,200× g for 15 min) or further purified utilizing Zymo-III RNA columns according to manufacture's instructions (Zymo). RNA was extracted from five bees collected from the colony entrance for each of the time-course samples.

### Arthropod Pathogen Microarray design and synthesis

Design principles used for APM oligonucleotides (70 nt) were based on previous pan-viral microarrays using ArrayOligoSelector (AOS) [54]. Briefly, array oligonucleotides were selected for uniqueness against an insect nucleic acid background, for ∼50% GC content to maintain high complexity, and for cross-reactivity of highly-conserved nucleic acid features with evolutionarily related targets (<−50 kcal/mol predicted binding energy). Arthropod pathogen oligonucleotides (GEO GPL11490) were synthesized by Invitrogen, suspended at 40 pmol/µL in 3× SSC and 0.4 pmol/µL control oligo and printed on poly-L-lysine slides (Thermo) with silicon pins as previously described [100]. Each oligonucleotide and its reverse complement were printed twice for redundancy. Arrays were allowed to air-dry and stored and room temperature. Prior to use, oligonucelotides were cross-linked to slides via UV exposure (600 mJ), washed with 3× SSC/0.2% SDS and blocked using a methylpyrrolidone solution (335 mL 1-methyl-2-pyrrolidinone, 5.5 grams succinic anhydride, 15 mL 1 M sodium borate).

### Sample Preparation for Arthropod Pathogen Microarray

*(Reverse Transcription, CyDye Labeling, Hybridization, Scanning)*

For each sample, 5 µL (∼15 µg nucleic acid) of extracted material was randomly primed and amplified as previously described [44], [45]. Briefly, an adapter-linked random nonamer (5′GTTTCCCACTGGAGGATANNNNNNNNN) was used to prime the reverse transcription reaction using SuperScript II (Invitrogen). The same oligo is used for two rounds of second-strand synthesis with Sequenase (USB) in order to produce adapter-flanked sequences from both RNA and DNA starting material. One-quarter of the random priming reaction is used in a 50 µLTaq PCR reaction for 25 cycles with a single primer (5′GTTTCCCACTGGAGGATA). One-tenth of the amplified material was further amplified for 10–20 cycles with a Cy3-linked primer (5′Cy3 -GTTTCCCACTGGAGGATA). Samples were purified with the Zymo DNA Clean and Concentrator (Zymo) and resuspended in a buffer of 3× SSC, 50 mM HEPES and 0.5% SDS, and hybridized on the APM overnight at 65°C. Arrays were washed and scanned with an Axon 4000A scanner. Samples were analyzed manually and scored as positive for a pathogen if at least three unique oligonucleotides hybridized with at least five times background intensity. Arrays were further analyzed by a second unbiased method using the E-Predict algorithm [54], [55], wherein all virus genomes were computationally hybridized to the array oligos and array results are compared to expected binding profiles. The top 5 unique oligos were removed and the algorithm reiterated twice in order to improve detection of low titer target(s) during a co-infection. Known honey bee pathogens were called positive if they exceeded a similarity score of 0.001 and were the highest ranked call in any iteration. In the event of a disagreement between the two analysis methods, a specific PCR reaction was performed, using material from the first PCR step, to resolve the call.

### Assessment of Arthropod Pathogen Microarray sensitivity

In order to estimate the sensitivity of the arthropod pathogen microarray (APM) two positive control samples were prepared in the presence and absence of pathogen-free honey bee RNA. A full-length (9,264 nucleotide) *Drosophila* C virus (DCV) clone was *in vitro* transcribed, serially diluted into honey bee RNA, reverse-transcribed, amplified, dye-labeled and hybridized to the APM as described above. Detection of at least 3 of the 8 unique DCV oligonucleotides and their reverse complements resulted in an estimated DCV detection level of 1.9×10^5^ genome copies (1 pg DCV genomic RNA) in an *A. mellifera* RNA (1 µg) background. Similarly, detection of a BQCV genome segment (452 nt), corresponding to one array oligo and its reverse complement, diluted into either pathogen-free honey bee RNA (0.5 µg) or water indicated detection limits of 1.2×10^5^ genome segment copies (30 fg BQCV RNA segment) and 1.2×10^4^ genome segment copies (3 fg BQCV RNA segment) respectively.

### PCR

Reaction conditions for polymerase chain reaction (PCR) amplifications of select samples were performed under the following conditions: 5 µL of 1∶10 dilution of PCR-amplified DNA and 10 pmol of each forward and reverse primers were amplified with Taq polymerase with the following cycling conditions: 95°C for 5 min; 95°C for 30 s, 50–60°C for 30 s, 72°C for 1 min, 35 cycles; final elongation 72°C for 7 min, hold at 4°C. Select samples were Sanger sequenced directly from ExoI and SAP treated PCR product or from colony PCR of TOPO cloned (Invitrogen) gel-extracted bands. Bands produced by PCR assays for known honey bee pathogens were sequenced until each molecular weight product was unambiguously associated with either a true positive or non-target amplification of the honey bee genome or microbiome. All PCR results for the four novel viruses were confirmed by Sanger sequencing.

### Quantitative PCR (qPCR)

qPCR was performed on pooled samples from each month. Equivalent amounts of RNA (10 µg) from each hive sample (monitor hives 1–20) were pooled according to the month in which they were collected (April 2009 to January 2010). Pooled RNA was further purified using Qiagen RNAeasy columns, including on column DNase Treatment (Qiagen). cDNA synthesis reactions were performed with SuperScriptIII (Invitrogen) according to manufacturer's instructions. In brief, RNA from each pooled sample (5 µg), random hexamer (1.25 µg) and dNTPs (0.5 mM each) were combined in a 50 µL reaction volume, incubated at 65°C (5 min), cooled on ice (1 min) and subsequently combined with 50 µL of 2× First-Strand Buffer containing SSIII (1000 U), DTT (5 mM), and RNaseOUT (200 U). Reverse transcription reactions were incubated for 12 hours at 42°C followed by inactivation of the reaction (70°C, 15 min). qPCR was performed in triplicate wells using 2 µL of cDNA as template in 20 µl reactions composed of HotStartTaq 2× Mastermix (Denville), 1× SYBR Green (Invitrogen), MgCl_2_ (3 mM), and forward and reverse primers (600 nM each) (Table S2) on a LightCycler480 (Roche). The qPCR thermo-profile consisted of a single pre-incubation 95°C (10 min), 35 cycles of 95°C (30 s), 60°C (30 s), and 72°C (30 s). No RT control reactions using pooled RNA as the template for qPCR were performed in triplicate on each plate. Target qPCR amplicons were cloned into pGEM-T (Promega) or TOPO CR 2.1 (Invitrogen) vectors and sequence verified. Plasmid standards, containing from 10^9^ to 10^2^ copies per reaction, were used as qPCR templates to assess primer efficiency and generate the pathogen-specific standard curves used to quantify the viral genome or rRNA copy number. The linear standard equations generated by plotting the crossing point (Cp) versus the log_10_ of the initial plasmid copy number for each primer set were as follows: BQCV Cp = −5.67×+59.44, R^2^ = 0.975; SBV Cp = −5.34×+56.33, R^2^ = 0.976; ABPV Cp = −4.03×+43.7, R^2^ = 0.995; LSV1 Cp = −4.21×+46.56, R^2^ = 0.993; LSV2 Cp = −3.66×+40.76, R^2^ = 0.998; ALP-Br Cp = −2.91×+34.76, R^2^ = 0.980; BSRV Cp = −3.28×+36.93, R^2^ = 0.999; *Nosema ceranae* Cp = −7.03×+69.43, R^2^ = 0.975; *Crithidia* rRNA Cp = −3.13×+36.44, R^2^ = 0.994 (LightCycler 480 Software, Abs Quant/2^nd^ Derivative Max, high sensitivity mode, Roche). The detection limits of each qPCR primer set were as follows: *Crithidia* and ALP-Br −10^2^ copies, LSV2 and BSRV −10^3^ copies, BQCV, SBV, ABPV, LSV1 and *Nosema* −10^4^ copies. Specific qPCR amplicons had Cp values of <30. Pathogen copy number data were reported per RT-qPCR reaction (Figure 4). Values obtained from the no RT control reactions, all below the detection limit of the assays, were subtracted from the total pathogen copy number for each month. An estimate of the number of viral genomes per bee can be obtained by multiplying the reported qPCR copy number values by 500. This estimate is based on the following: typical RNA yield was approximately 50 µg per bee, each qPCR reaction was performed on cDNA generated from 100 ng RNA, therefore each well represents 1/500^th^ of an individual bee. We choose to represent the raw data, since each monthly-pooled sample was composed of variable bee numbers due to differential sampling frequency each month. In addition, qPCR with a host primer set, *Apis m.* Rpl8, was performed using 1 µL cDNA template on each qPCR plate to ensure consistency and cDNA quality. qPCR products were analyzed by melting point analysis and 2% agarose gel electrophoresis (Figure S1).

### LSV Northern Blot

Honey bees from two LSV positive honey bee colonies were homogenized in 500 µL phosphate buffered saline (PBS, UCSF Cell Culture Facility) with a sterile zinc-coated steel ball bearing (5 mm) using a TissueLyzer II (Retsch) for 4 minutes at 30 Hz; 5 bees per micro-centrifuge tube. Lysates were centrifuged for 10 minutes at 12,000× g and RNA was extracted from both the supernatant and the bee carcass containing pellet using TRIzol Reagent (Invitrogen) according to manufacturer's instructions. RNA was further purified using RNAeasy columns, including on column DNase Treatment (Qiagen). RNA (15 µg per lane) was combined with glyoxal-based loading dye (Northern-Max® sample loading dye, Ambion) and denatured at 50°C for 30 min prior to gel electrophoresis on an ethidium bromide containing 1.5% agarose BPTE gel using BPTE running buffer. BTPE buffer is composed of 10 mM PIPES, 30 mM Bis-Tris, 1 mM EDTA, pH 6.5. The gel was imaged using a UV lightbox and then soaked in 0.05 N NaOH for 20 min prior to overnight transfer to a membrane using the Nytran® SuPerCharge Turboblotter™ system and 20× SSC. Following transfer the membrane was washed in 2× SSC (2×5 min), dried and UV crosslinked using a Stratalinker (Stratagene). LSV specific primers were used to amplify sequences corresponding approximately to the 5′, middle, and 3′ regions of the viral genome (primers listed in Table S2). The PCR products were column purified using the MinElute Reaction Cleanup Kit (Qiagen), labeled with {á^32^P} dCTP using Ready-To-Go™ DNA Labelling Beads (-dCTP) (Amersham; GE Healthcare) and used as LSV-specific Northern blot probes. The membrane was cut into three pieces and incubated, while rotating, in ULTRAhyb buffer (Ambion) at 42°C for 30 min prior to the addition of the radiolabled probes (10^6^ counts per minute per mL hybridization buffer). Following overnight hybrization at 42°C, the membranes were washed at 42°C (2×5 min in 2× SSC 0.1% SDS; 2×15 min in 1× SSC 0.1% SDS, 2×15 min in 0.1× SSC 0.1%SDS). Phosphoimaging was performed using a Typhoon 9400 imager (GE Healthcare) (Figure S4).

### Negative strand-specific RT-PCR

LSV strain 1 and 2 positive samples were analyzed for the presence of negative-strand RNA, which is indicative of virus replication, using strand-specific RT-PCR [39], [64], [65]. RNA from select samples (*e.g.* pooled July sample) was further purified using Qiagen RNAeasy columns, including on column DNase Treatment (Qiagen). cDNA synthesis reactions were performed with SuperScriptIII (Invitrogen) according to manufacturer's instructions using negative strand-specific LSV1 and 2 primers tagged with an additional 21 nt of sequence (5′- GGCCGTCATGGTGGCGAATAA) at their 5′ end [65]; the tag sequence shares no homology with LSV nor to the honey bee genome (primer sequences listed in Table S2). In brief, RNA from each sample (1 µg), tagged-negative strand specific LSV primer (10 pmole) or random hexamers (50 ng) and dNTPs (0.5 mM each) were combined in a 10 µL reaction volume, incubated at 65°C (5 min), cooled on ice (1 min) and subsequently combined with 10 µL of 2× First-Strand Buffer containing SSIII (200 U), DTT (5 mM), and RNaseOUT (40 U). Reverse transcription reactions were incubated for 1 hour at 50°C followed by inactivation of the reaction (70°C, 15 min). Unincorporated primers present in the RT reactions were digested with exonuclease I (Fermentas), 0.1 Units per reaction which corresponds to a 10-fold excess of enzyme relative to the initial primer concentration, at 37°C for 30 min followed by heat inactivation at 80°C for 15 minutes. PCR was performed using 2 µL of exonuclease I treated cDNA template in 25 µl reactions containing 10 pmol each of a tag-specific forward primer (TAGS) and an LSV-specific reverse primer using the following cycling conditions: 95°C for 5 min; 95°C for 30 s, 58°C for 30 s, 72°C for 30 s, 35 cycles; final elongation 72°C for 4 min, hold at 4°C. In addition to amplification and detection of the LSV replicative form using tagged-negative strand primed cDNA template and TAGS forward and LSVU-R1717 PCR primers, negative and positive controls were performed (Figure S5 – labeled (1)). Negative controls included utilizing unprimed RT reaction as a template for PCR amplification using TAGS forward and LSVU-R1717 primers (labeled (2)), LSV tagged negative-strand primed cDNA template in PCR reaction in which only the LSVU-R1717 primer was added in order to ensure that all of the unincorporated RT primer was digested with exonuclease I and thus not involved in priming the PCR reaction (labeled (5)), and no template PCR using LSV qPCR primer sets (labeled (6)). Positive controls included using random hexamer primed cDNA as template for PCR amplification using LSV1 or LSV2 -specific forward primer and LSVU-R-1717 (labeled (3)) and random hexamer primed cDNA amplified using LSV-specific qPCR primer sets (labeled (6)). PCR products were analyzed using agarose (2%) gel electrophoresis (Figure S5).

### *Crithidia mellificae* strain SF - Microscopy, Culturing and DNA Purification

Honey bees were collected from a San Francisco, CA (U.S.A.) colony previously identified to be *Crithidia* positive by microarray and PCR testing. Honey bees were immobilized by chilling at 4°C for 20 minutes, briefly washed in 70% ethanol, and decapitated prior to dissection. The SF strain was isolated from honey bee intestines dissected in a sterile environment, minced and placed in a T25 flask and cultured in BHT medium composed of Brain Heart Infusion (BHI) 28.8 g/L (DIFCO), tryptose 4.5 g/L (DIFCO), glucose 5.0 g/L, Na_2_HPO_4_ 0.5 g/L, KCl 0.3 g/L, hemin 1.0 mg/L, fetal bovine serum (heat inactivated) 2% v/v, pH 6.5, and containing penicillin G sodium (10^6^ units/L) and streptomycin sulfate (292 mg/L) at 27°C [101]. Free active *Crithidias* were observed 24 hours post inoculation. Parasites were maintained by subculture passage every 4 days; stable liquid nitrogen stocks were archived. Light microscopy of live parasites was performed using a Leica DM6000 microscope equipped with Hamamatsu C4742-95 camera and Volocity Software (PerkinElmer). Imaging fixed parasites (4% paraformaldehyde, 20 min) facilitated visualization of DAPI (4′,6-diamidino-2-phenylindole) stained nuclear and kinetoplast DNA. Images of fixed *Crithidia mellificae* were obtained using both the Leica DM6000 microscope and a Zeiss LSM 510-M microscope equipped with both a 63× objective numerical aperture 1.4, and a 100× objective numerical aperture 1.4.

For DNA purification, *Crithidia mellificae* (∼10^6^ trypanosomes/mL culture medium) were pelleted by centrifugation (800×g for 6 min) and washed with PBS prior to DNA extraction. DNA was extracted using the DNeasy Genomic DNA Extraction Kit (Qiagen) as per the manufacturer's instructions. Bees from Crithidia positive hives were homogenized by TissueLyser as above and DNA extracted using the DNeasy kit for the initial PCR screens, after suspension in either PBS or 1× Micrococcal Nuclease Buffer (NEB).

### Ultra Deep Sequencing Library Preparation

Total nucleic acid from all twenty monitor hives at time-point 17 (August 5, 2009) was pooled (approximately 3 µg per hive). One quarter was treated with RNase A/T1 (Fermentas) and genomic DNA was isolated using a DNeasy column (Qiagen). 50 ng of genomic DNA was prepared for deep sequencing by Nextera recombinase (Epicentre) per the manufacturer's instructions. The remaining nucleic acid was treated with Turbo DNase (Ambion) and column purified (Zymo) before being split into thirds. One third was enriched for mRNAs with dT-linked Dynabeads (Invitrogen). RNA from this fraction and from a second unenriched fraction were primed for RT and second-strand synthesis with an adapter linked oligo as above using oligo SolCommonN (5′CGCTCTTCCGATCTNNNNNN). The third fraction of RNA was primed with an anchored oligo dT and subjected to two rounds of second strand synthesis with SolCommonN. Half of the initial material was amplified with primer SolCommon (5′CGCTCTTCCGATCT) with KlenTaq (Sigma) at an annealing temperature of 37°C for 20 cycles. Reactions were cleaned by Zymo column, analyzed by NanoDrop spectrophotometer and 50 ng was used in a four-primer PCR reaction. In a 50 µL KlenTaq reaction, 10 pmol each of primers 5Sol1 (5′AATGATACGGCGACCACCGA) and 5Sol1 (5′CAAGCAGAAGACGGCATACG) and 0.5 pmol of Sol1 (5′AATGATACGGCGACCACCGAGATCTACACTCTTTCCCTACACGACGCTCTTCCGATCT) and Sol2 (5′CAAGCAGAAGACGGCATACGAGATCGGTCTCGGCATTCCTGCTGAACCGCTCTTCCGATCT) were incubated for 2 cycles annealing at 37°C and 10 cycles at 55°C. Products were run on an 8% native acrylamide TBE gel (Invitrogen) and a 300–350 nt smear was cut out and electro-eluted. The product was further amplified at an annealing temperature of 55°C with primers 5Sol1 and 5Sol2 for 5–10 cycles until at least 30 ng of material was produced, as determined by NanoDrop. Libraries were sequenced on an Illumina Genome Analyzer II with a V3 cluster generation kit and V5 sequencing reagent as per the manufacturer's instructions, producing paired-end 65 nt reads.

### Solexa Data Analysis and Virus Genome Recovery

Six pools of sequence data were downloaded from GenBank: *Nosema ceranae* (draft genome), *Spiroplasma* (*S. citri* draft genome and all sequences longer that 500 nt), DNA viruses of arthropods (all complete genomes), all small RNA viruses of arthropods except *dicistroviridae* and *iflavirus* (complete genomes), all members of *dicistroviridae* and iflavirus except those infecting honey-bees (complete genomes), and all known honey bee RNA viruses (complete genomes). Each pool was converted into a Blast library and queried against the entire Solexa dataset by BlastN and tBlastx. Hits with an e-value greater than 1×10^−3^ were extracted along with their paired end, regardless of similarity. Each pool was assembled using the Geneious sequence analysis package [102]. Contigs greater than 250 nt were queried again against the dataset by tBlastx with an e-value threshold of 1×10^−5^. Any positive hits were then queried against the NR database with the same parameters to eliminate spurious hits.

Contigs that appeared divergent or that were derived from non-honey bee associated viruses were extended using the entire read dataset using a paired-end contig extension algorithm (“PRICE” Graham Ruby, manuscript under preparation, software available at http://derisilab.ucsf.edu). The extended contigs were then independently confirmed by PCR recovery and Sanger sequencing. Individual paired-end reads that were discordant with the recovered contigs were used to further nucleate new contigs via contig extension. Primer3 [103] was used to design primers bridging adjacent contigs, as determined by mapping onto known virus genomes. Individual viruses or other microbes were queried with a BlastN threshold e-value of 1×10^−7^ (W7) to determine read counts.

### Statistical Analysis

Associations were calculated treating each hive sample at each time-point as a distinct event. P-values (Chi-square values) and odds ratios (OR) listed were calculated by the OpenEpi statistical package v2.3 (http://www.openepi.com/OE2.3/Menu/OpenEpiMenu.htm). Only seven microbes with incidences in the study set of at least 10% (20 incidences in 197 samples) were examined for association, resulting in 28 discrete association tests and the corresponding Bonferroni multiple testing correction. Microbes occurring infrequently were not used in association tests and so did not contribute to multiple testing correction.

### Data Availability and Compliance with Standards

APM design and results have been submitted to GEO (design accession GPL11490 and array data accession GSE28235) and are MIAME compliant. All Sanger sequence-confirmed deep sequencing assemblies have been submitted to GenBank (accessions listed in text).

## Supporting Information

Figure S1 **Gel electrophoresis of RT-qPCR products from pooled-monthly samples.** qPCR products were amplified using the primer sets listed in Table S2: *Nosema ceranae* 249 bp, *Crithidia mellificae* 153 bp, black queen cell virus (BQCV) 141 bp, sacbrood virus (SBV) 103 bp, acute bee paralysis virus (ABPV) 177 bp, Lake Sinai Virus strain 1 (LSV1) 153 bp, Lake Sinai Virus strain 2 (LSV2) 225 bp, Aphid Lethal Paralysis Virus Strain Brookings (ALP-Br) 141 bp, and Big Sioux River virus (BSRV) 281 bp. Molecular weight ladder (L), April 2009 (A), May (M), June (J6), July (J7), August (A), September (S), October (O), November (N), December (D), January 2010 (J1); RNA no RT control (−), plasmid standard copy number (10^X^).
(TIFF)

Figure S2 **Dicistrovirus Phylogeny.** Dicistrovirus IRES elements were aligned by ClustalW and a Neighbor-Joining tree generated by the Geneious Tree Builder (100 replicates). IAPV – Israeli acute paralysis virus (NC009025), KBV – Kashmir bee virus (NC004807), ABPV – acute bee paralysis virus (NC002548), SINV1 – *Solenopsis invicta* virus 1 (NC006559), TSV – *Taura* syndrome virus (NC003005), ALPV – acute lethal paralysis virus (NC004365), ALPV strain Brookings (Q871932), RhPV – *Rhopalosiphum padi* virus (NC001874), BSRV – Big Sioux River virus (JF423195-8), CrPV – cricket paralysis virus (NC003924), DCV – *Drosophila* C virus (NC001834), TV – *Triatoma* virus (NC003783), HPV – *Himetobi P* virus (NC003782), PSV – *Plautia Stali* intestine virus (NC003779), HCV – *Homalodisca coagulata* virus (NC008029), and BQCV – black queen cell virus (NC003784); red text – common honey bee viruses; blue text – novel viruses.
(TIFF)

Figure S3 **RT-PCR results from pooled-monthly samples.** (A) *Nosema apis* (268 bp), (B) deformed wing virus (DWV; 194 bp), (C) *Apocephalus borealis* (phorid fly; 500 bp), (D) *Apis mellifera* ribosomal protein L8 (Rpl8; 100 bp). Molecular weight ladder (L), April 2009 (A), May (M), June (J6), July (J7), August (A), September (S), October (O), November (N), December (D), January 2010 (J1); RNA only no RT control (−), water (H_2_O), and positive control (+).
(TIFF)

Figure S4 **Detection of the LSV genome by denaturing 1.5% agarose gel electrophoresis and Northern blots using three LSV-specific probes.** RNA (15 µg) extracted from the supernatants of homogenized honey bees was transferred to a membrane and probed using LSV-specific probes corresponding to different regions of the genome (P1 – 1482–1744, P2 – 2289–2477, and P3 – 4509–4714) as described in Materials and Methods.
(TIFF)

Figure S5 **Detection of the replicative form of LSV1 and LSV2 by negative strand-specific RT-PCR.** The pooled July RNA sample was analyzed for the presence of LSV negative-strand RNA, which is indicative of virus replication, using strand-specific RT-PCR as described in Materials and Methods; RT-PCR products from reactions were analyzed by agarose (2%) gel electrophoresis.
(TIF)

Figure S6 ***Crithidia mellificae*** **, strain SF movies.** Light microscopy of live parasites was performed using a Leica DM6000 microscope (100× objective) equipped with Hamamatsu C4742-95 camera and Volocity Software (PerkinElmer).
(MP4)

Figure S7 ***Crithidia mellificae*** **, strain SF movies.** Light microscopy of live parasites was performed using a Leica DM6000 microscope (100× objective) equipped with Hamamatsu C4742-95 camera and Volocity Software (PerkinElmer).
(MP4)

Table S1 Arthropod pathogen microarray results from test samples.
(DOCX)

Table S2 Primers used in this study, * denotes primer sets used for PCR screening results in Figure 3B, ** denotes qPCR primer sets used to obtain the results in Figure 4 and Figure S3.
(DOCX)
