# Supplementary figures and images for: Temporal Analysis of the Honey Bee Microbiome Reveals Four Novel Viruses and Seasonal Prevalence of Known Viruses, Nosema, and Crithidia
Source: PLoS One. 2011 Jun 7;6(6):e20656. doi: 10.1371/journal.pone.0020656 (PMC3110205; doi:10.1371/journal.pone.0020656)

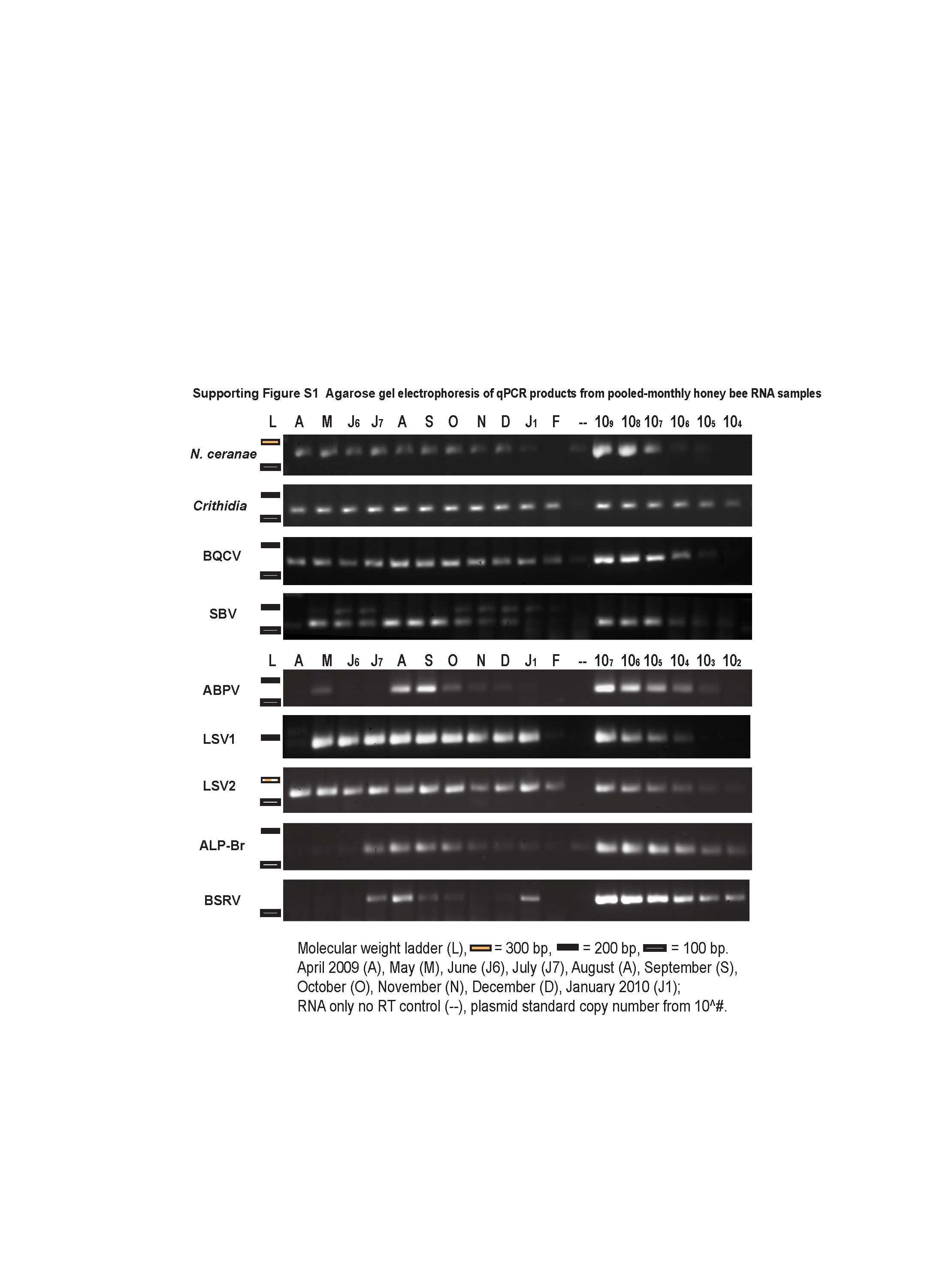

Supplement: Figure S1 — Gel electrophoresis of RT-qPCR products from pooled-monthly samples. qPCR products were amplified using the primer sets listed in Table S2: Nosema ceranae 249 bp, Crithidia mellificae 153 bp, black queen cell virus (BQCV) 141 bp, sacbrood virus (SBV) 103 bp, acute bee paralysis virus (ABPV) 177 bp, Lake Sinai Virus strain 1 (LSV1) 153 bp, Lake Sinai Virus strain 2 (LSV2) 225 bp, Aphid Lethal Paralysis Virus Strain Brookings (ALP-Br) 141 bp, and Big Sioux River virus (BSRV) 281 bp. Molecular weight ladder (L), April 2009 (A), May (M), June (J6), July (J7), August (A), September (S), October (O), November (N), December (D), January 2010 (J1); RNA no RT control (−), plasmid standard copy number (10X). (TIFF) [file pone.0020656.s001.tif]

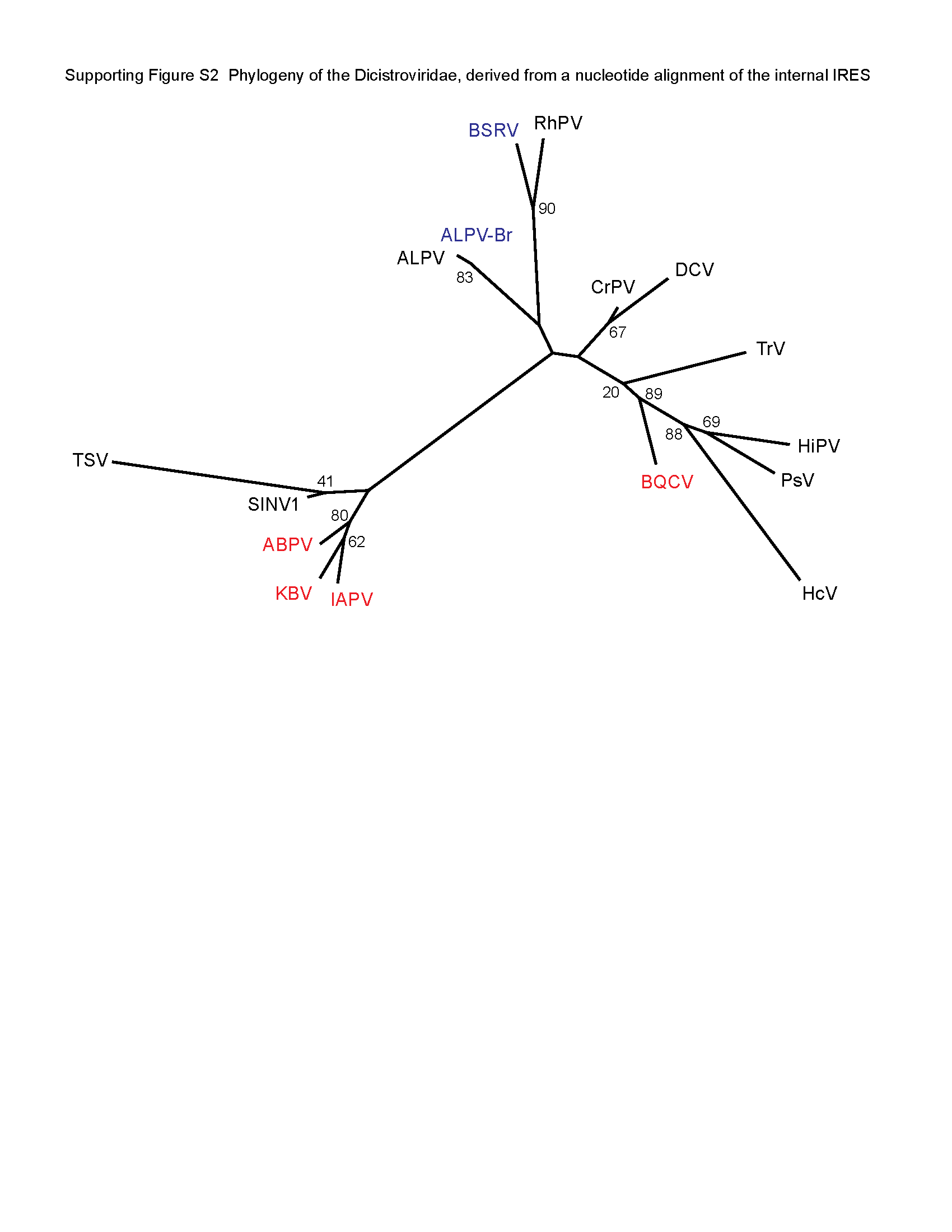

Supplement: Figure S2 — Dicistrovirus Phylogeny. Dicistrovirus IRES elements were aligned by ClustalW and a Neighbor-Joining tree generated by the Geneious Tree Builder (100 replicates). IAPV – Israeli acute paralysis virus (NC009025), KBV – Kashmir bee virus (NC004807), ABPV – acute bee paralysis virus (NC002548), SINV1 – Solenopsis invicta virus 1 (NC006559), TSV – Taura syndrome virus (NC003005), ALPV – acute lethal paralysis virus (NC004365), ALPV strain Brookings (Q871932), RhPV – Rhopalosiphum padi virus (NC001874), BSRV – Big Sioux River virus (JF423195-8), CrPV – cricket paralysis virus (NC003924), DCV – Drosophila C virus (NC001834), TV – Triatoma virus (NC003783), HPV – Himetobi P virus (NC003782), PSV – Plautia Stali intestine virus (NC003779), HCV – Homalodisca coagulata virus (NC008029), and BQCV – black queen cell virus (NC003784); red text – common honey bee viruses; blue text – novel viruses. (TIFF) [file pone.0020656.s002.tif]

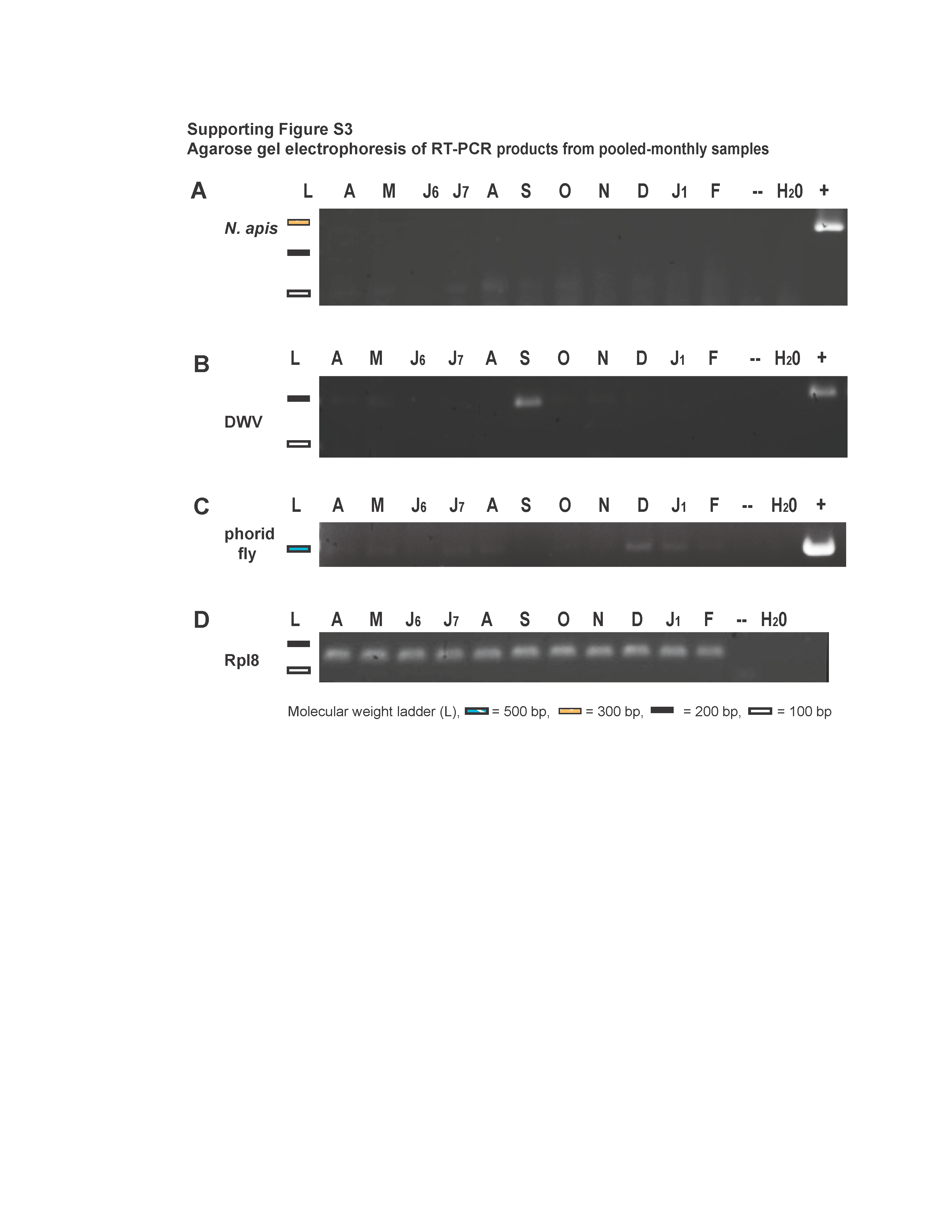

Supplement: Figure S3 — RT-PCR results from pooled-monthly samples. (A) Nosema apis (268 bp), (B) deformed wing virus (DWV; 194 bp), (C) Apocephalus borealis (phorid fly; 500 bp), (D) Apis mellifera ribosomal protein L8 (Rpl8; 100 bp). Molecular weight ladder (L), April 2009 (A), May (M), June (J6), July (J7), August (A), September (S), October (O), November (N), December (D), January 2010 (J1); RNA only no RT control (−), water (H2O), and positive control (+). (TIFF) [file pone.0020656.s003.tif]

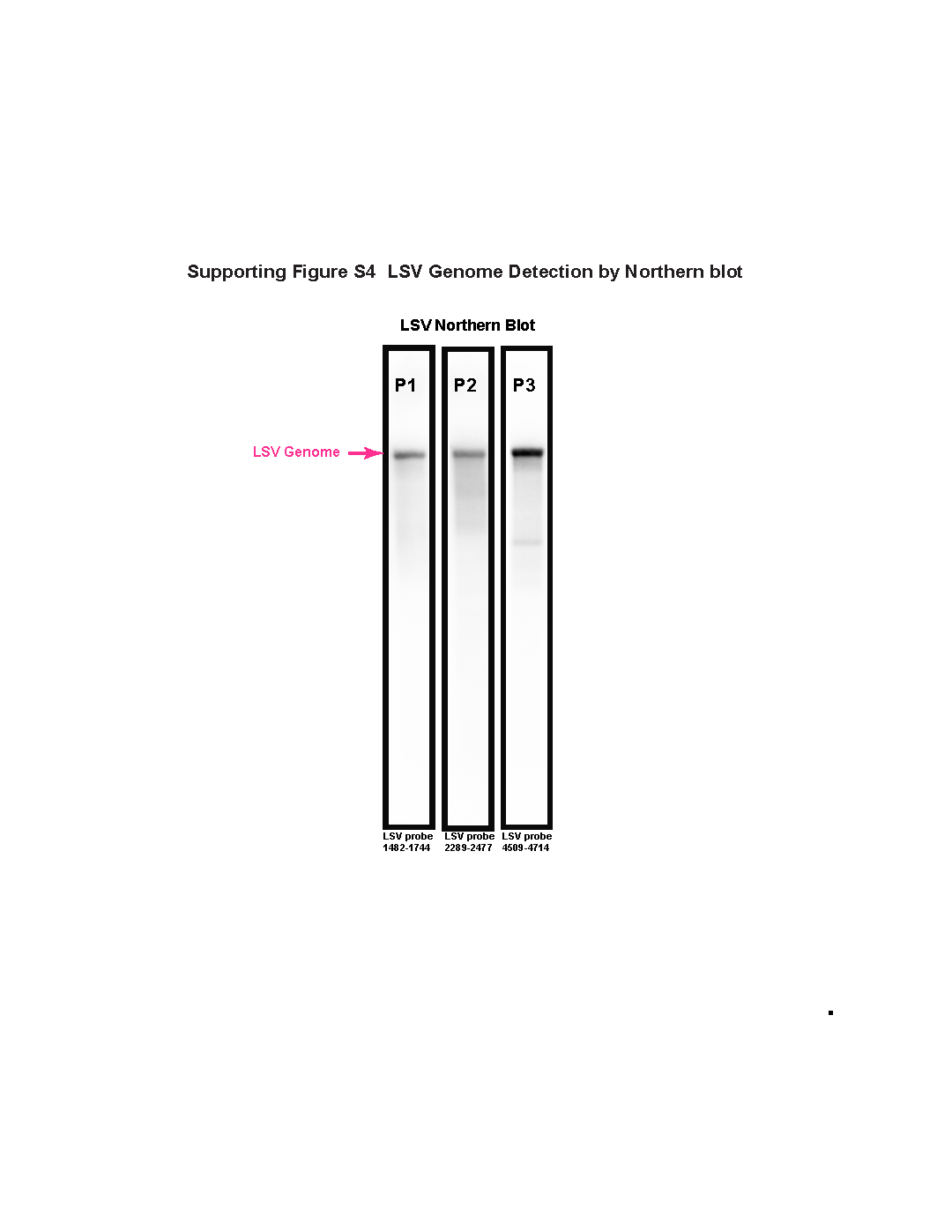

Supplement: Figure S4 — Detection of the LSV genome by denaturing 1.5% agarose gel electrophoresis and Northern blots using three LSV-specific probes. RNA (15 µg) extracted from the supernatants of homogenized honey bees was transferred to a membrane and probed using LSV-specific probes corresponding to different regions of the genome (P1 – 1482–1744, P2 – 2289–2477, and P3 – 4509–4714) as described in Materials and Methods. (TIFF) [file pone.0020656.s004.tif]

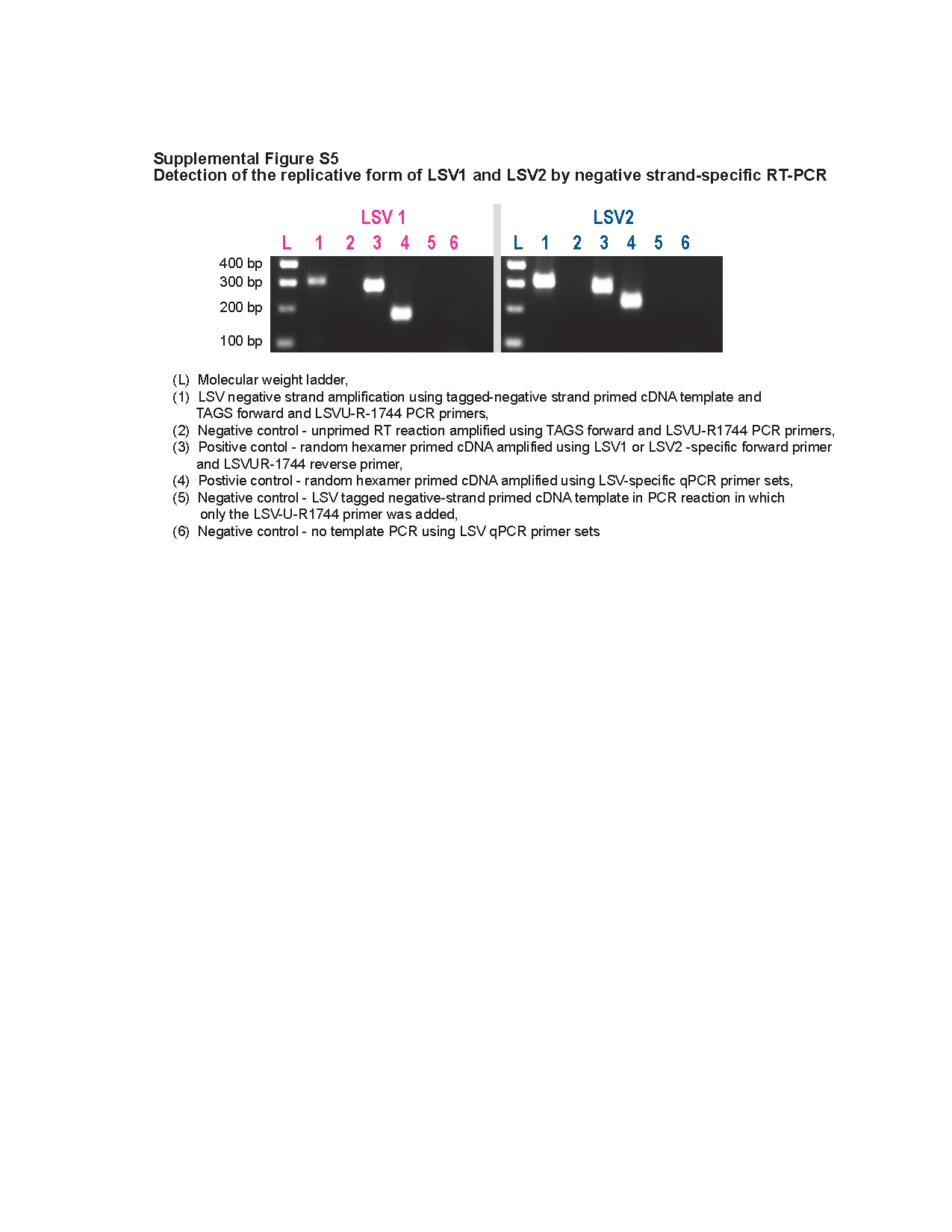

Supplement: Figure S5 — Detection of the replicative form of LSV1 and LSV2 by negative strand-specific RT-PCR. The pooled July RNA sample was analyzed for the presence of LSV negative-strand RNA, which is indicative of virus replication, using strand-specific RT-PCR as described in Materials and Methods; RT-PCR products from reactions were analyzed by agarose (2%) gel electrophoresis. (TIF) [file pone.0020656.s005.tif]
