# Supplementary material for: Temporal Analysis of the Honey Bee Microbiome Reveals Four Novel Viruses and Seasonal Prevalence of Known Viruses, Nosema, and Crithidia
Source: PLoS One. 2011 Jun 7;6(6):e20656. doi: 10.1371/journal.pone.0020656 (PMC3110205; doi:10.1371/journal.pone.0020656)
Supplement: Table S1 — Arthropod pathogen microarray results from test samples. (DOCX) [file pone.0020656.s008.docx]

**Supporting Table S1. Arthropod Pathogen Microarray test samples**

| **Sample** | **Arthropod Pathogen Microarray Results** |
| --- | --- |
|  |  |
| **Overwinter Collapse (2007; MT)** | BQCV, DWV, IAPV, KBV |
|  |  |
| ***Apis mellifera* (CA)** | SBV |
|  |  |
| ***Varroa destructor*** | *Varroa*, DWV |
|  |  |
| ***Apis mellifera*** (drone; CA) | BQCV, DWV |
|  |  |
| ***Apis mellifera***  (Hive 53; CA - House Bees) | BQCV, KBV |
| ***Apis mellifera***  (Hive 53; CA - Foragers) | BQCV |
|  |  |
| ***Apis mellifera***  (Hive 42; CA - House Bees) | BQCV |
| ***Apis mellifera***  (Hive 42; CA - Foragers) | BQCV |
|  |  |
| ***Bombus sp.* (CA)** | *Crithidia, Varroa* |
|  |  |
| ***Apis mellifera***  (Hive UCD; CA - Hairless Bee) | CBPV |
| ***Apis mellifera***  (Hive UCD; CA - Healthy Bee) | *Crithidia* |
|  |  |
| ***Vespula* sp.**  (Hive SM, CA, Feb. 2009) | ABPV, DWV, *Crithidia* |
| ***Apis mellifera***  **(**Hive SM, CA, Feb. 2009) | ABPV, DWV, BQCV, *Nosema* |
| ***Varroa destructor***  (Hive SM, CA Feb. 2009) | *Varroa*, DWV |
|  |  |
| **Bee Bread** (Healthy Hive Fall 2009) | positive controls |
| **Bee Bread**  (CCD-affected Hive 1,Winter 2008/9**)** | DWV |
| **Bee Bread**  (CCD-affected Hive 2,Winter 2008/9) | DWV, VDV-1 |
|  |  |
| **Overwinter Collapse (2010; OK)** | DWV, SBV, *Nosema* |
| **Overwinter Collapse (2010; OK)** | BQCV, *Nosema* |
|  |  |
