# Supplementary material for: Temporal Analysis of the Honey Bee Microbiome Reveals Four Novel Viruses and Seasonal Prevalence of Known Viruses, Nosema, and Crithidia
Source: PLoS One. 2011 Jun 7;6(6):e20656. doi: 10.1371/journal.pone.0020656 (PMC3110205; doi:10.1371/journal.pone.0020656)
Supplement: Table S2 — Primers used in this study, * denotes primer sets used for PCR screening results in Figure 3B, ** denotes qPCR primer sets used to obtain the results in Figure 4 and Figure S3. (DOCX) [file pone.0020656.s009.docx]

**Supporting Table S2. Primers used in this study**

| **Genome/Gene** | **NCBI Accession No.; GI No.** | **Primer Name** | **Sequence (5'-3')** | **Product Size (bp)** | **Reference** |
| --- | --- | --- | --- | --- | --- |
|  |  |  |  |  |  |
| **Ribosomal Protein L8 (*Apis m.*)** | XM_393671.3 GI:110760460 | Rpl8Fw | TGGATGTTCAACAGGGTTCATA | 100 | Evans et al. (2006) Insect Mol Bio |
|  |  | Rpl8Rev | CTGGTGGTGGACGTATTGATAA |  |  |
|  |  |  |  |  |  |
| **acute bee paralysis virus (ABPV)** | NC_002548.1 GI:10314009 | ABPV-F-8125 | AGCCACTATGTGCTATCGTAT | 206 | Grabensteiner et al. (2007) J Invert Path |
|  |  | ABPV-R-8331 | ATGGTGACCTCTGTGTCATTA |  |  |
|  |  |  |  |  |  |
|  |  | qABPV-F-5457* | GGATGAGAGAAGACCAATTG | 177 | Highfield et al. (2009)  Appl Environ Micro |
|  |  | qABPV-R-5634* | CCAATCTTGGGAATAAACATTAGTTC |  | this work |
|  |  |  |  |  |  |
| **black queen cell virus (BQCV)** | NC_003784.1 GI:20451021 | qBQCVorf2F_6664* | TCCTCAAATCTGGAGCGAAC | 141 | this work |
|  |  | qBQCVorf2R_6805* | GTATTCGCTGGCCGTAAAAC |  |  |
|  |  |  |  |  |  |
|  | NC_003784.1 GI:20451021 | BQCV-F-7850 | TGGTCAGCTCCCACTACCTTAAAC | 700 | Benjeddou et al. (2001) Appl Environ Micro |
|  |  | BQCV-R-8550 | GCAACAAGAAGAAACGTAAACCAC |  |  |
|  |  |  |  |  |  |
| **chronic bee paralysis virus (CBPV)** | NC_010711.1 GI:188543025 | CBPV-F_2580 | AGTTGTCATGGTTAACAGGATACGAG | 454 | Ribiere et al. (2002) Apidologie |
|  |  | CBPV-R_3034 | TCTAATCTTAGCACGAAAGCCGAG |  |  |
|  |  |  |  |  |  |
| **deformed wing virus (DWV)** | AY292384.1 GI:31540603 | DWV-F-1165 | CTTACTCTGCCGTCGCCCA | 194 | Chen et a. (2005) J Invert Path |
|  |  | DWV-R-1338 | CCGTTAGGAACTCATTATCGCG |  |  |
|  |  |  |  |  |  |
| **Israeli acute paralysis virus (IAPV)** | NC_009025.1 GI:126010924 | IAPV-F-8860 | AGACACCAATCACGGACCTCAC | 474 | Maori et al (2007) J Gen Virol |
|  |  | IAPV-R-9334 | AGATTTGTCTGTCTCCCAGTGCACAT |  |  |
|  |  |  |  |  |  |
| **Kashmir bee virus (KBV)** | NC_004807.1 GI:30793779 | KBV-F-5406 | GATGAACGTCGACTATTGA | 412 | Stoltz et al. (1995) J Apicult Res |
|  |  | KBV-R-5818 | TGTGGGTTGGCTATGAGTCA |  |  |
|  |  |  |  |  |  |
|  |  |  |  |  |  |
| **sacbrood virus (SBV)** | AF092924.1 GI:4416206 | SBV-F-4958 | GCTGAGGTAGGATCTTTGCGT | 823 | Chen et al. (2004) J Invert Path |
|  |  | SBV-R-5781 | TCATCATCTTCACCATCCGA |  |  |
|  |  |  |  |  |  |
|  | AF092924.1 GI:4416206 | SBV-F-3103** | GGTGTCTAACTTTTATGGACCACCA | 103 | Cox-Foster et al. (2007) Science |
|  |  | SBV-R-3206** | CCTTTTCTATGCTATCATCCATCTGA |  |  |
|  |  |  |  |  |  |
| **aphid lethal paralysis virus strain Brookings (ALP-Br)** | Q871932 | qALP-Br F-2680** | ACACCATAGTTCGCGAAGAACGCA | 141 | this work |
|  |  | qALP-Br R 2821** | GCAGCACCGGAAACGTTTTTATGG |  |  |
|  |  |  |  |  |  |
|  |  | ALP-Br-F-2936* | AACGTCGTATGCTACGATGAACTCG | 464 | this work |
|  |  | ALP-Br-R-3400* | GGGTTAAATTCAATTCCAGTACCACGG |  |  |
|  |  |  |  |  |  |
| **Big Sioux River virus (BSRV)** |  | qBSRV-F-235 | GCGCCTATTTTCTGCAGCGCC | 281 | this work |
|  |  | qBSRV-R-546 | CCCGCGATATAATTGCGTTTGTGAGC |  |  |
|  |  |  |  |  |  |
|  |  | BSRV_4714F* | RGTGCAGCTTTATGCGTTGCC | 519 | this work |
|  |  | BSRV_37R* | CCGCTGTTGAGAATAAGGATATCCAGG |  |  |
|  |  |  |  |  |  |
| **Lake Sinai virus (universal)** |  | qLSVU-F-2350 | TTATCTCGCGCCGCCACCTC | 188 | this work |
|  |  | qLSVU-R-2538 | AGAGGGTACCGCGACACCCATG |  |  |
|  |  |  |  |  |  |
|  |  | LSV1&2U-F-4481 | CTTGAGACTCAGGGATTCGTCAC | 205 | this work |
|  |  | LSV1&2U-R-4686 | AGGGACGACGGAGCACAATT |  | this work |
|  |  |  |  |  |  |
| **Lake Sinai virus strain 1 (LSV1)** | HQ871931 | qLSV1-F-2515** | AGAGGTTGCACGGCAGCATG | 153 | this work |
|  |  | qLSV1-R-2668** | GGGACGCAGCACGATGCTCA |  |  |
|  |  |  |  |  |  |
|  |  | LSV1-F-2294* | TTATCTCGCGCCGCCACCTC | 672 | this work |
|  |  | LSV1-R-2966* | ATCGCCGCTGCAACGTGACC |  |  |
|  |  |  |  |  |  |
| **Lake Sinai virus strain 2 (LSV2)** | HQ888865 | qLSV2-F-1783** | CGTGCTGAGGCCACGGTTGT | 225 | this work |
|  |  | qLSV2-R-2008** | GCGGTGTCGATCTCGCGGAC |  |  |
|  |  |  |  |  |  |
|  |  | LSV2-F-3954* | CGGCCGGTCTAGCGTGGTTG | 558 | this work |
|  |  | LSV2-R-4512 | TGGCAAGCTGTGACGAATCCCT |  |  |
|  |  | |  |  | |
|  | Primers for Negative strand PCR  (Numbered according to LSV2 seq.) | |  |  | |
|  |  | LSVU-F-1483-TAGS | **GGCCGTCATGGTGGCGAATAA**  GACTTCATCATCCATCTGTGCGA | 255 | this work |
|  |  | LSV1-F-1434-TAGS | **GGCCGTCATGGTGGCGAATAA**  CAGGTGCAGAGCAATTGGATTCA | 304 | this work |
|  |  | LSV2-F-1434-TAGS | **GGCCGTCATGGTGGCGAATAA**  TAGGTGTCGGGCCATAGGGTTTG | 304 | this work |
|  |  | **TAGS Forward** | **GGCCGTCATGGTGGCGAATAA** |  | Plaskon et al. (2009) PLoS One |
|  |  |  |  |  |  |
|  |  | LSVU-R-1717 | CCATATCATAAGTTGGCAAGTG |  | this work |
|  |  | LSVU-F-1483 | GACTTCATCATCCATCTGTGCGA | 234 | this work |
|  |  | LSV1-F-1434 | CAGGTGCAGAGCAATTGGATTCA | 283 | this work |
|  |  | LSV2-F-1434 | TAGGTGTCGGGCCATAGGGTTTG | 283 | this work |
|  |  |  |  |  |  |
|  |  |  |  |  |  |
| ***Nosema sp.*** | DQ235446.1 GI:7810022 | Nosema pan rRNA F-322 | GGCAGTTATGGGAAGTAACA | 207 | Chen et al. (2008) J Inv Path |
|  |  | Nosema pan rRNA R-529 | GGTCGTCACATTTCATCTCT |  |  |
|  |  |  |  |  |  |
| ***Nosema ceranae*** | DQ673615.1 GI:110293152 | Ncer 16S rRNA F-274 | CAGTTATGGGAAGTAATATTATATTG | 73 | Cox-Foster et al. (2007) Science |
|  |  | Ncer 16S rRNA R-347 | TTGATTTGCCCTCCAATTAATCAC |  |  |
|  |  |  |  |  |  |
|  | DQ486027.1 GI:94962170 | Nosema ceranae F-4186** | CGGATAAAAGAGTCCGTTACC | 249 | Chen et al. (2008) J Inv Path |
|  |  | Nosema ceranae R-4435** | TGAGCAGGGTTCTAGGGAT |  |  |
|  |  |  |  |  |  |
| ***Nosema apis*** | U97150.1 GI:2988413 | NosApis-F-4395 | CCATTGCCGGATAAGAGAGT | 379 | Chen et al. (2008) J Inv Path |
|  |  | NosApis-R-4774 | CACGCATTGCTGCATCATTGAC |  |  |
|  |  |  |  |  |  |
|  | U97150.1 GI:2988413 | NosApis-F-4395 | CCATTGCCGGATAAGAGAGT | 268 | Chen et al. (2008) J Inv Path |
|  |  | NosApis-R-4663 | CCACCAAAAACTCCCAAGAG |  |  |
|  |  |  |  |  |  |
| ***Crithidia mellificae*** |  | CF_TrypanF1 | GTTGACGGAATCAACCAAACAAAT | 715 | Cox-Foster et al. (2007) Science |
|  |  | CF_TrypanR1 | GCGTCAGAGGTGAAATTCTTAGACC |  |  |
|  |  |  |  |  |  |
|  |  | qCrFw1** | TCCACTCTGCAAACGATGAC | 153 | this work |
|  |  | qCrRev1** | GGGCCGAATGGAAAAGATAC |  |  |
|  |  |  |  |  |  |
|  |  | Crith_GAPDH | TTYGCCGYATYGGYCGCATGG | 802 | Hamilton et al. (2004) Int  J Parasitol |
|  |  |  | GTTYTGCAGSGTCGCCTTGG |  |  |
|  |  |  |  |  |  |
| ***Spiroplasma sp.*** |  | Spiroplasma_271_F28 | CGCAGACGGTTTAGCAAGTTTGGG | 271 | Liang et al. (2010) Lett Appl Microbiol |
|  |  | Spiroplasma_271_R5 | AGCACCGAACTTAGTCCGACAC |  |  |
|  |  |  |  |  |  |
| ***A. borealis*** |  | Phorid_rRNA 1F | GTACACCTATACATTGGGTTCGTACATTAC | 500 | this work |
|  |  | Phorid_rRNA 1R | GAGRGCCATAAAAGTAGCTACACC |  |  |
| *** End-point PCR screening primers used in this work** | | | | | |
| **** qPCR primers used in this work** | | | | | |
